# Reactions to (the absence of) control and workplace arrangements: experimental evidence from the internet and the laboratory

**DOI:** 10.1007/s10683-020-09666-8

**Published:** 2020-08-13

**Authors:** Katrin Schmelz, Anthony Ziegelmeyer

**Affiliations:** 1 University of Konstanz, PO Box D 131, 78457 Konstanz, Germany; 2 Thurgau Institute of Economics, Hafenstrasse 6, 8280 Kreuzlingen, Switzerland; 3 Queen’s Management School, Queen’s University Belfast, Belfast, Northern Ireland, UK

**Keywords:** Hidden benefits of abstaining from control, Laboratory, Internet, Workplace arrangements, C81, C90, C93, M52

## Abstract

This paper reports an experiment designed to assess the influence of workplace arrangements on the reactions to (the absence of) control. We compare behavior in an Internet and a laboratory principal-agent game where the principal can control the agent by implementing a minimum effort requirement. Then the agent chooses an effort costly to her but beneficial to the principal. Our design captures meaningful differences between working from home and working at the office arrangements. Online subjects enjoy greater anonymity than lab subjects, they interact in a less constrained environment than the laboratory, and there is a larger physically-oriented social distance between them. Control is significantly more effective online than in the laboratory. Positive reactions to the principal’s choice not to control are observed in both treatments, but they are significantly weaker online than in the laboratory. Principals often choose the highest control level, which maximizes their earnings.

**Electronic supplementary material:**

The online version of this article (10.1007/s10683-020-09666-8) contains supplementary material, which is available to authorized users.

## Introduction

During the early months of 2020, the number of people working from home dramatically increased as a result of the lockdown measures implemented to tackle the spread of Covid-19. With all but key workers confined to their homes, the virtual office became the new norm in many countries around the globe.^1^ As a result, working patterns could shift permanently as many companies made substantial investments to make remote work possible and workers might not want to return to the office for the entire week.

Like other flexible scheduling and work arrangements, working from home (WFH) challenges existing managerial approaches designed for office employees since such approaches might be inadequate to supervise and elicit performance from distant employees. To deal with the lack of direct oversight, employers could either develop supervisory relations based on trust and autonomy or they could turn to tougher supervisory procedures. Whether WFH calls for a different managerial approach heavily depends on whether the nature of the employment relationship, close or distant, influences work motivation or employees’ reactions towards (the absence of) supervision.

Bruno S. Frey and coauthors have repeatedly argued that the closer the relationship between employers and employees the more likely controlling reduces work effort and performance (Frey 1993, 1997; Frey and Jegen 2001). Close employment relationships foster the intrinsic motivation of employees, and as a consequence an intervention of the employer perceived to be controlling is likely to crowd out work motivation. In distant employment relationships intrinsic motivation is less present and crowding out plays little role. The imposition of tougher controlling on distant employees is therefore less likely to backfire on employers. Frey’s hypothesis suggests that employers should control office and WFH employees differently and adjust to the lack of day-to-day personal oversight in WFH arrangements with new ways of tightly controlling work. This perspective contrasts with the advice commonly offered by management consultants to supervisors of WFH employees according to which the management of these employees is best secured by an emphasis on trust rather than close regulation. However, interview-based evidence has questioned this oversimplified recommendation (Felstead et al. 2003). At the present time, researchers and practitioners are still debating about the most effective way to manage distant employees (Lautsch et al. 2009; Groen et al. 2018).

This paper reports an experiment designed to assess the influence of workplace arrangements on the reactions to (the absence of) control. We compare behavior in an Internet and a laboratory implementation of a principal-agent game where the principal can control the agent by imposing either a low or a medium effort level before the agent chooses an effort costly to her but beneficial to the principal. Our principal-agent game is a straightforward extension of the laboratory game used by Falk and Kosfeld (2006) (henceforth FK) in their main treatments. In FK’s low (respectively medium and high) control treatment, the principal chooses the lower bound $\underline{x} \in \{0, 5\}$ (respectively $\{0, 10\}$ and $\{0, 20\}$) of the agent’s set of efforts. The agent then chooses an effort $x \in \{\underline{x}, \underline{x} + 1, \ldots , 120\}$. FK find that principals earn more if they choose to trust ($\underline{x} = 0$) than if they choose to control ($\underline{x} > 0$). These differences are statistically significant in the low and medium control treatments but not in the high control treatment. Enforcing a minimum effort backfires largely because many agents exert less effort if the principal controls rather than trusts them. The adverse effect of control on many agents’ performance has been replicated in subsequent studies (Ziegelmeyer et al. 2012; Burdin et al. 2018).

To better mimic naturally-occurring agency relationships, we extend the basic one-shot principal-agent game implemented by FK in two major ways. First, we implement a repetitive trial environment where subjects are informed about the payoff consequences of their choices after each repetition of the game which allows them to learn through personal experience and reflection. However, we match our subjects according to the ‘no contagion’ protocol so that no agent’s behavior in a given round can affect the behavior of a principal the agent is paired with at a later round (Kamecke 1997). Indeed, our study aims at shedding light on the reciprocal motives underlying agents’ reactions to (the absence of) control in the two treatments. By suppressing repeated-game incentives for agents to act in the interest of the principal, this matching protocol prevents reciprocity considerations from being confounded with repeated-game considerations. Second, rather than fixing the control level exogenously, we allow the principal to impose either a low or a medium effort level before the agent chooses an effort. This extension of the principal-agent game better reflects the exertion of managerial control in the field. Typically, employers not only choose whether or not to control their employees, but they also choose to which extent they exert control. Moreover, this extension enables us to distinguish between the categorical effect of control and the marginal effect of variations in control.^2^

Another extension of our design is that subjects guess the average behavior of their counterpart. For each control level, principals guess the average effort of agents whereas agents guess the percentage of principals choosing the respective control level. If the principal believes that the effectiveness of control is more strongly undermined by agents’ reciprocal motives in the laboratory than online, he should implement lower control levels in the laboratory than online. Thus, the beliefs of payoff-maximizing principals determine how much control they enforce and the extent to which these beliefs are correct influences how effective the enforced control level is. Agents’ beliefs, on the other hand, may not influence strongly their efforts since they move second and react to the control level chosen by the principal. Still, according to some of the theoretical models that explain FK’s evidence, agents’ beliefs are key determinants of their efforts. We outline in Sect. 2.2 these theoretical arguments.

Compared to the laboratory setting, three distinctive features of the Internet setting may cause subjects’ behavior to differ in the two treatments. First, online subjects are paired across different locations and enjoy greater subject-subject anonymity than lab subjects who are recruited to a single location and see each other before entering the agency relationship.^3^ Accordingly, lab subjects might consider the possibility of running into their counterparts after the session and engage in reputation-building behavior.^4^ This could enhance the propensity of lab agents to act reciprocally and could also lead lab principals to trust their agents. Second, nearly all aspects of the laboratory setting, including physical features and even the possibility of getting up and doing something else, are determined by the experimenter. The Internet setting, on the other hand, offers many more alternative activities and distractions as online subjects make their interactive choices at their place of choice (most likely home). The fact that online subjects complete their tasks in a less constrained environment than lab subjects implies that online agents have higher autonomy outside of the agency relationship which might lead them to accept more easily a reduced choice autonomy in the agency relationship. Third, online principal-agent pairs are physically disconnected contrary to lab pairs which interact in the same location. Charness et al. (2007) explore the effect of increasing the physically-oriented social distance on trust and reciprocity and they find that the fraction of choices indicating positive reciprocity varies inversely with the social distance. This finding suggests that we could observe higher levels of managerial control and lower reciprocal reactions to (the absence of) control in the Internet than in the laboratory treatment. Hereafter, we simply refer to agency relationships as being more distant over the Internet than in the laboratory which encompasses greater subject-subject anonymity, a less constrained environment, and a larger physically-oriented social distance.

The study provides four main findings. First, efforts increase with the control level in each treatment. Still, control is significantly more effective in the Internet than in the laboratory treatment. We therefore confirm Frey’s hypothesis even when the distance in agency relationships is weakly manipulated. Second, reciprocal motives drive agents’ behavior in both treatments, but reciprocity is significantly weaker online than in the laboratory. Third, in the absence of control, agents exert significantly less effort online than in the laboratory. Agents’ behavior suggests the existence of hidden benefits of abstaining from control which are stronger in the laboratory than in the Internet treatment.^5^ Finally, in both treatments principals often choose the highest control level, which maximizes their earnings.

### Limitations of our study

Our study is a first attempt at exploring experimentally the influence of workplace arrangements on the effectiveness of control. Inevitably, there are several differences between the home and office environments that are left out in our design. The extent to which these omitted differences matter for the effectiveness of control in the two workplace arrangements remains an open empirical question and is likely to be job-specific. First, we follow FK by assuming that the agent’s effort is perfectly observable which implies that there is no difference regarding the level of oversight about effort provision in the two treatments. As rightly argued by Bloom et al. (2015), there is a range of service jobs, such as sales, IT support, and secretarial assistance, for which the link between effort and performance is direct and such jobs are particularly suitable for telecommuting. Still, future economic experiments should consider alternative settings where the principal receives an imperfect signal of the agent’s effort so that the level of oversight about effort provision can vary between the home and office treatments. Second, employers perceive office employees closer to them than WFH employees not only because of their physical proximity per se but also because this physical proximity leads to more frequent interactions which in turn generates some emotional proximity. In this sense, close and distant agency relationships in Frey’s hypothesis might be better understood as interpersonal and impersonal agency relationships. Clearly, our experiment uses a weak manipulation of the distance in agency relationships. This weak manipulation is, however, a useful starting point since emotional proximity can easily be incorporated into future experiments (see our discussion of the related literature below). Third, different from workplace arrangements in the field, our subjects are randomly assigned to the laboratory and Internet conditions. In contrast, if a firm supports WFH, employees can typically choose whether they want to make use of it (with the exception of the natural experiment triggered by the Covid-19 pandemic). In some firms, office work is even the default and the permission to work from home might increase the employee’s intrinsic motivation. We exclude such potential mechanisms in our design because allowing subjects to choose between workplace arrangements creates a confound. Agents who self-select into WFH schemes might differ in other characteristics from those who cannot choose and the effect of distance per se would be difficult to infer. Arguably, the differences between the home and office environments omitted in our design imply that if we observe a greater effectiveness of control online than in the lab then work motivation is also less likely to be reduced by managerial control in WFH arrangements.

Another concern of our manipulation is that online data might be of limited quality due to a lower degree of scrutiny compared to the laboratory (Anderhub et al. 2001). Recent experimental studies conclude that online-based and lab-based inferences are equally reliable provided that similar procedures are used in both settings (Arechar et al. 2018; Hergueux and Jacquemet 2015; Normann et al. 2014). As detailed in Appendices A and B of the supplementary material, we invested much effort to address this concern and relied on the same subject pool and similar procedures in both treatments. We compare the quality of our online and laboratory data in Appendix G of the supplementary material and we conclude that data of similar quality were collected in the two treatments.

### Related literature

Our experimental study relates to two strands of the literature.

First, a small experimental literature investigates the impact of workplace arrangements on individual productivity. Dutcher (2012) evaluates the WFH environmental effects on productivity in creative and dull individual tasks. Like in our experiment, half the subjects completed the tasks in the laboratory and the other half outside the laboratory. The results indicate that completing the tasks outside the laboratory affects positively the productivity of creative tasks but negatively the productivity of dull tasks. Bloom et al. (2015) conduct a field experiment with call center employees in a Chinese company. Half of the employees invited to the study volunteered to WFH and the other half kept working at the office. WFH lead to a significant increase in the employees’ performance. We are not aware of a former study that investigates the impact of workplace arrangements on the effectiveness of control and our experimental study fills this gap.

Second, three experimental studies analyze the influence of the nature of the agency relationship on agents’ work motivation.^6^ Masella et al. (2014) test the impact of group identity on the effectiveness of managerial control. Subjects are first assigned to different, artificially created, groups. The authors induce group identity using subjects’ painting preferences and they enhance it by having subjects participate in, among others, a quiz where groups compete while group members communicate. Subjects then interact in a principal-agent game (almost) identical to FK’s. The results show that the effectiveness of control is comparable in between-group and within-group agency relationships though the mechanisms for how control is perceived are group-specific. Riener and Wiederhold (2016) also test the effect of group norms on the effectiveness of control in FK’s principal-agent game (except that the principal’s choice set is extended to three control levels). They compare a group-building treatment where subjects initially play a coordination game to gain common experience (CE) with an autarky treatment where subjects complete a task in isolation (NCE). They observe that agents’ effort is crowded out more strongly in the CE than in the NCE treatment. The fact that their group induction task facilitates gaining positive experience among group members and mutual judgment about this group experience might explain why, contrary to Masella et al. (2014), their manipulation impacts agents’ reactions to (the absence of) control. Dickinson and Villeval (2008) contrast impersonal agency relationships where subjects are matched as strangers and the anonymity of principal-agent pairs is preserved with interpersonal agency relationships where subjects are matched as partners and the pairs’ anonymity is removed (each pair is allowed to engage in five-minutes of face-to-face social interaction). The agent is engaged in a real-effort task which is likely to generate substantial intrinsic motivation and the principal chooses the probability with which the agent’s output is audited. The authors find that the disciplining effect of monitoring dominates the crowding-out effect in both agency relationships and that tighter monitoring by the principal crowds out the agent’s effort only in interpersonal agency relationships. Our experiment complements this second strand of the literature which considers a different manipulation of the distance in agency relationships. Future economic experiments could combine our manipulation WFH versus working at the office with induced group identity or interpersonal relationships to increase our understanding of the impact of workplace arrangements on the effectiveness of control.

Section 2 outlines our experimental design and procedures, and it provides detailed research hypotheses. Section 3 reports our results and Sect. 4 concludes. The online supplementary material contains seven appendices with, among others, the experimental instructions and complementary statistical analyses.

## Experimental design, procedures, and hypotheses

We first present the principal-agent interaction that forms the basis of our experimental design. This first subsection details the players’ choice sets and the monetary payoffs that result from the interaction. Second, we outline our experimental design and procedures, and we provide detailed research hypotheses.

### A principal-agent interaction of managerial control

Consider an agent who engages in a productive activity which is costly to her but beneficial to the principal. Before the agent exerts effort, the principal can either decide to leave the agent’s effort set unrestricted by choosing “no control” ($\underline{e} = 1$) or he can decide to restrict the agent’s effort set by choosing one of two control levels: “low control” ($\underline{e} = 2$) or “medium control” ($\underline{e} = 3$). The agent then chooses an effort level $e \in \{\underline{e}, \underline{e} + 1, \ldots , 10\}$. Table 1 shows the monetary payoffs (in experimental currency units) where the fair and most efficient effort level locates slightly above the middle ($e = 7$).

**Table 1:** Monetary payoffs by effort level

	Effort level									
	1	2	3	4	5	6	7	8	9	10
Agent’s monetary payoffs	99	98	96	93	89	83	75	65	51	35
Principal’s monetary payoffs	1	16	29	41	53	64	75	82	87	90

Several considerations led to the players’ monetary payoffs shown in Table 1. First, exerting more effort than the minimal one is cheap for the agent and extremely beneficial for the principal. Thus, the choice of controlling induces small monetary costs for the agent and large direct benefits for the principal. However, the fact that control induces small monetary costs for agents is precisely the reason why those willing to act in the principal’s interest are likely to interpret the choice of controlling as a strong signal of distrust. Similarly, the fact that control induces large direct benefits for principals makes it likely for agents to interpret the choice of not controlling as a strong signal of trust. Consequently, we expect to observe strong reactions to (the absence of) control in our experiment. Second, effort costs are assumed to be convex since exerting low effort at work is usually not very costly but once the agent is working to capacity marginal effort costs become tremendous. Third, benefits from the agent’s effort are assumed to be concave which reflects productivity losses due to physical restrictions.

Our setting extends FK’s principal-agent game by allowing the principal to impose either a low or a medium effort level before the agent chooses an effort (a similar extension is present in Riener and Wiederhold, 2016). This feature better reflects the exertion of managerial control in the field and it enables us to observe how agents react to the categorical and marginal effects of control. Still, even under medium control the restriction of the agent’s effort set is rather mild. Together with our payoff parameters, this implies that the disciplining effects of control are weak in our setting. We chose these parameters to ensure that agents can always exert a much larger effort than the minimum requirement by the principal so that abstaining from control has the potential to be profitable.

### Design

In both treatments subjects repeatedly take part in the principal-agent interaction described in Sect. 2.1 where the payoffs in Table 1 are in experimental currency units. We employ the strategy method, meaning that the agent makes her choice for each of the three control levels before knowing the principal’s actual decision.^7^ Concretely, each agent is asked to choose a triplet of effort levels $\left( e(1), e(2), e(3)\right)$ where $e(1) \in \{1, 2, \ldots , 10\}$ is payoff-relevant in case the principal does not enforce a minimal effort, $e(2) \in \{2, 3, \ldots , 10\}$ is payoff-relevant in case the principal enforces a low effort, and $e(3) \in \{3, 4, \ldots , 10\}$ is payoff-relevant in case the principal enforces a medium effort.

In a given session, each subject is assigned either the role of agent or the role of principal. Subjects gain experience with the context and the behavior of others during 10 repetitions of the interaction. Roles are kept constant over all rounds. The matching follows a ‘no contagion’ protocol so that no agent’s behavior in a given round can affect the behavior of a principal the agent is paired with at a later round.^8^ Though a partner-matching design would better mimic naturally occurring employment relationships, it would provide repeated-game incentives for agents to act in the interest of the principal. We suppress repeated-game incentives as we aim at shedding light on the nature of the agents’ reciprocal motives in our two treatments. Our matching protocol prevents these potential reciprocity considerations from being confounded with repeated-game considerations.

#### Belief elicitation

Before they interact in the agency relationship, subjects are asked to guess the average behavior of their counterpart. In each round, subjects make three guesses. Principals are asked to guess, for each control level, the average effort that will be chosen by agents (since we employ the strategy method, for each control level *all* agents choose an effort). Each principal reports his guesses by keying in a vector $\left( b_P(1), b_P(2), b_P(3)\right)$ with $\underline{e} \le b_P({\underline{e}}) \le 10$. Agents are asked to guess, for each control level, the natural frequency of principals that will choose the respective control level. Each agent reports her guesses by keying in a vector $\left( b_A(1), b_A(2), b_A(3)\right)$ with $0 \le b_A({\underline{e}}) \le 100$ and $b_A(1) + b_A(2) + b_A(3) = 100$.

We expect principals’ beliefs to have a large impact on the levels of control they choose to enforce. Indeed, if a payoff-maximizing principal believes that the effectiveness of control is more strongly undermined in the laboratory than online, then he should implement lower control levels in the laboratory than online. Agents’ efforts, on the other hand, may be less affected by their beliefs. At the intuitive level, how strongly the agent believes that the principal will choose a certain control level should have little impact on her effort as she reacts to the control level chosen by the principal. Still, Sliwka (2007) provides a theoretical explanation for the crowding out of work motivation that emphasizes the role played by agents’ beliefs (the same logic holds true in Ellingsen and Johannesson, 2008). As sketched below, in this theoretical setting, agents’ guesses about the average behavior of principals affect their reactions to (the absence of) control.

Sliwka (2007) proposes that some agents are conformists who react positively to the absence of control if and only if they believe that most of the non-conformist agents are willing to act in the principal’s interest. When the absence of control is a credible signal that most non-conformist agents can be trusted, conformists exert less effort if controlled than if not controlled. According to this model, conformists’ efforts follow from their beliefs about the proportion of fair-minded non-conformists and these beliefs are shaped by the level of control that principals choose to enforce. In other words, conformists view principals’ choices as signals about the norm of behavior of non-conformists. It is therefore reasonable to assume that, when asked to guess the average behavior of principals, conformists would form beliefs about the non-conformists’ norm of behavior. Conformists would then update these beliefs based on the control levels chosen by principals.

#### Feedback

We limit the possibility to learn about the choices of other subjects. Once all guesses and choices have been made in a given round, subjects are only informed about the behavior of their counterpart. Subjects do not learn about the correctness of their guesses during the experimental session.^9^

#### Earnings

Subjects receive two types of payments. First, each subject is paid a flat amount of 30 experimental currency units (ECUs) for completing a survey. Second, subjects are paid for their performance in the interactive part of the experiment. Concretely, one of the 10 repetitions of the principal-agent game is randomly selected at the end of the session (each round is equally likely to be selected). Then, another random draw selects either the choice made in the principal-agent interaction or one of the three guesses as the basis for the subject’s payment. The randomly chosen guess is paid according to the following scheme: If an agent’s (principal’s) guess differs by no more than 5 percentage points (0.5 effort levels) from the true value then the subject earns 70 ECUs. Otherwise the subject earns 20 ECUs.

### Practical procedures

Both treatments were conducted with the help of an Internet platform developed by the authors. All 440 subjects were students who had agreed to participate in economic experiments. The data were collected in two waves. Two sessions per treatment were first conducted in Jena with 106 students from the University of Jena (November 2010 and January 2011). We then conducted another six sessions per treatment in Konstanz with 334 students from the University of Konstanz (November 2014 and April 2015). All sessions followed exactly the same procedures.^10^

In each location we divided randomly the subject pool into two parts. Half of the subjects was invited to the laboratory treatment and the other half was invited to the online treatment. Subjects were invited using the ORSEE recruitment system (Greiner 2015). Subjects invited to the laboratory treatment were not informed of the online treatment and vice versa. If they had been aware of the online treatment, some agents in the laboratory treatment may have lowered their efforts because, contrary to the online agents, they had to travel to the laboratory. In a similar vein, if they had been aware of the laboratory treatment, some agents in the online treatment may have increased their efforts because, contrary to the lab agents, they could stay at home.

In each treatment students received an invitation email with a link to a registration page. On this page they were informed about the general rules of the study, and about the fact that the other participants are those they usually interact with in the laboratory. For registration students had to enter some information (gender, month and year of birth, nationality, mother tongue, and email address). Each student could register only once. Registered subjects received a survey token via email. Answering the survey questions took on average 10 min and subjects had a time frame of a few days to do so. In each treatment subjects completed the survey at their place of choice (e.g. at home).^11^

Subjects who completed the survey could register for an experimental session and received a session token to the experiment via email. To circumvent a potential impact of the survey on choices in the interactive part experimental sessions were conducted on a later day. Each session took slightly more than one hour.

In the online treatment there was a prearranged start time for each of the eight sessions which took place in the afternoon or evening, and each of the 232 subjects had to log on not later than that time. Like for the survey, subjects made their interactive choices at their place of choice. The eight sessions of the laboratory treatment took place in the afternoon or evening with a total of 208 subjects. Instructions were not read aloud.

In both treatments subjects received their earnings in the laboratory. In the online treatment subjects were informed that they would receive a compensation fee for collecting their earnings which corresponds to the usual show-up fee in the two locations (2.50 euros in Jena and 3 euros in Konstanz). In the laboratory treatment we also added the usual show-up fee to subjects’ earnings.^12^ In Jena, 1 ECU was converted to 0.15 euros. To adjust for differences in purchasing power, absolute earnings were slightly raised in Konstanz where 1 ECU was converted to 0.20 euros. Subjects in Jena (Konstanz) earned 15.42 (19.60) euros on average (about 21 (22) US dollars at the time of the sessions).^13^ Further details about the participation process are provided in Appendix B, and Appendix D.1 shows the main screens of the experiment (translated to English), in particular the instructions.

#### The (likely) absence of a treatment-specific participation bias

Though students from the same subject pool were randomly assigned either to the online treatment or to the laboratory treatment, a treatment-specific participation bias might exist as it is arguably more convenient to participate at home than at the laboratory. If such a bias exists, we believe that it is minor for the following three reasons. First, alike subjects in the laboratory treatment, subjects in the online treatment had to register for an experimental session which took place on a given day and started at a given time. Thus, subjects in the online treatment faced the same schedule constraint as subjects in the laboratory treatment and they were aware of that fact before registering for the experiment. Second, both in Jena and in Konstanz, the laboratory is located at the university campus and therefore very easy for students to reach. Accordingly, coming to the laboratory is not necessarily associated with more effort than participating from home. In fact, we observe that the fraction of subjects who took part in the laboratory session they signed up for is almost identical to the fraction of subjects who took part in the online session they signed up for (see the second row of Table 2). Though they had several days to reconsider their decision to participate in the experiment, the fact that subjects needed to come to the laboratory did not impact negatively their willingness to fulfill their commitment. Third, Table 2 shows that the main demographic characteristics of students are very similar in the two treatments: the fraction of female subjects, the mean age, and the sample composition according to the academic major are highly comparable in the laboratory and Internet treatments.^14^

**Table 2:** Participation rate and characteristics of the students sample in each treatment

	Laboratory treatment	Internet treatment
Participation rate*	91%	92%
(conditional on signing-up for a session)		
Gender (% female)	52%	49%
Mean age (in years)	21.5	22.1
Academic major		
Business Administration & Economics	31%	34%
Other Behavioral & Social Sciences	28%	31%
Humanities	19%	18%
Engineering, Life & Natural Sciences	23%	18%

*Out of the subjects who registered for the survey, 97% (95%) in the laboratory (Internet) treatment completed it. Out of the subjects who completed the survey, 98% (99%) in the laboratory (Internet) treatment signed up for a session. Appendix C in the supplementary material details the participation rates over the entire course of the study

### Hypotheses

Our experiment mainly aims at assessing the influence of workplace arrangements on the reactions to (the absence of) control. We rely on the differences in effort levels to capture these reactions where the difference $e(2) - e(1)$ relates to the categorical effect of control and the difference $e(3) - e(2)$ relates to the marginal effect of control. Thus, the outcome variables to test our main research hypothesis are the differences $e(2) - e(1)$ and $e(3) - e(2)$. Our first and main research hypothesis is (a weak version) of Frey’s hypothesis.

#### **Hypothesis 1**

**[Effort differences]**. Differences in agents’ effort due to an increase in the level of control are larger online than in the laboratory.

Concretely, we postulate that the difference between the agent’s efforts under low control (*e*(2)) and under no control (*e*(1)) as well as the difference between the agent’s efforts under medium control (*e*(3)) and under low control (*e*(2)) are strictly larger in the Internet than in the laboratory treatment:

- $$e(2) - e(1) \vert _{Internet} \, > \, e(2) - e(1) \vert _{Laboratory}$$ ; and
- $$e(3) - e(2) \vert _{Internet} \, > \, e(3) - e(2) \vert _{Laboratory}$$.

We therefore expect that control is more effective (has less negative effects on the principal’s profit) in the Internet than in the laboratory treatment.

Along with testing Frey’s hypothesis, we aim at providing insight into the extent and nature of the behavioral motives underlying agents’ reactions to (the absence of) control. As already mentioned, former experiments on FK’s principal-agent game found that a sizeable fraction of agents exert more effort when the principal decides not to control than when he decides to control. To account for agents’ behavior, economists have focused primarily on reciprocity-based explanations (note that if agents care solely about the distribution of payoffs then their effort is independent of whether the principal controls them or not). FK argue that control can backfire because agents that are intrinsically motivated to act in the principal’s interest perceive control as a signal of distrust. Consequently, the agent reacts to control by choosing a lower effort than she would have chosen if the principal had decided not to control. FK’s interpretation of the agents’ reactions to control is therefore based on negative reciprocity which generates hidden costs of control.^15^

Burdin et al. (2018), on the other hand, argue that agents’ behavior should be interpreted in terms of positive reciprocity. The authors compare two treatments: one in which control can be exerted by a principal as in FK’s main treatments; and another in which it can be exerted by a third party who is given a show-up fee and does not directly benefit from the agent’s effort. They find that, in the absence of control, effort levels are significantly higher in the principal treatment than in the third party treatment. However, under control, effort levels are similar in the two treatments. Based on their findings, the authors conclude that intrinsically motivated agents reward with greater effort the principals that decide not to control, rather than punishing them with lower effort for the choice of controlling. The observation that some agents exert more effort in the absence of control than under control suggests the existence of hidden benefits of abstaining from control.

If negative reciprocity is the main driver of agents’ behavior, then we should observe similar efforts under no control in the two treatments and higher efforts under control in the Internet than in the laboratory. On the other hand, if positive reciprocity is the main driver of agents’ behavior, then we should observe lower efforts under no control in the Internet than in the laboratory and similar efforts under control in the two treatments. To provide evidence on the extent and nature of the reciprocal motives in our two treatments, we therefore decompose Hypothesis 1 into two sub-hypotheses. First, we postulate that agents’ reciprocal motives are weaker online than in the laboratory since the agency relationship is more distant in the Internet than in the laboratory treatment.

#### **Hypothesis 1a**

**[Reciprocity]**. Reciprocity is weaker online than in the laboratory.

An agent who reacts positively to the absence of control (or negatively to control) chooses a higher effort in the absence of control than if controlled. Still, to measure the magnitude of agents’ reciprocal motives accurately we have to exclude the disciplining effects of control. Following the procedure employed by FK, minimal efforts in the no control condition $(e(1) = 1)$ are set equal to 2 when comparing agents’ efforts in the low and no control conditions. Similarly, minimal efforts in the low control condition $(e(2) = 2)$ are set equal to 3 when comparing agents’ efforts in the medium and low control conditions. Reciprocity to low control implies that the difference between efforts under low control and the shifted efforts under no control $(e(2) - max[e(1), 2])$ is negative and reciprocity to medium control implies that the difference between efforts under medium control and the shifted efforts under low control $(e(3) - max[e(2), 3])$ is negative. Thus, Hypothesis 1a states that:

- $$e(2) - max[e(1),2] \vert _{Internet} \, > \, e(2) - max[e(1),2] \vert _{Laboratory} < 0$$; and
- $$e(3) - max[e(2),3] \vert _{Internet} \, > \, e(3) - max[e(2),3] \vert _{Laboratory} < 0$$.

Second, we shed light on the nature of reciprocal motives in our two treatments. In line with the evidence provided by Burdin et al. (2018), we expect agents’ behavior to be mainly driven by positive reciprocity. We therefore postulate that a more distant agency relationship in the Internet than in the laboratory treatment implies that the principal’s decision not to control is rewarded with lower effort online than in the laboratory.

#### **Hypothesis 1b**

**[Difference in efforts under no control]**. In the absence of control, agents exert less effort online than in the laboratory: $e(1) \vert _{Internet} \, < \, e(1) \vert _{Laboratory}$.

Finally, we conjecture that principals anticipate that control is more effective online than in the laboratory.

#### **Hypothesis 2**

** [Control level]**. Principals enforce larger control levels online than in the laboratory: $\underline{e} \vert _{Internet} \, > \, \underline{e} \vert _{Laboratory}$.

## Results

We observe that subjects’ behavior in Jena and Konstanz does not differ in any meaningful way. Indeed, we derived the p-values of 42 tests that compare subjects’ beliefs and choices in the two locations and we find that the distribution of these 42 p-values is very close to the uniform distribution (see Appendix E for more details). We therefore base our further statistical analysis on the pooled data of the two locations.

Below, we first report descriptive statistics on agents’ efforts for the different levels of control in the two treatments. Then we test formally our two research hypotheses.

### Descriptive statistics on agents’ reactions to (the absence of) control

Table 3 shows agents’ efforts as a function of the control level in the two treatments. In both panels the first row reports the average effort and the second row reports standard deviation followed by 1st quartile followed by median followed by 3rd quartile for each control level. We observe that the average effort in the absence of control is around the largest minimum effort enforceable by the principal, and that the average effort increases with the control level in each treatment.^16^ Still, the average effort under no control is larger in the laboratory than in the Internet treatment whereas increases in average effort are larger online than in the laboratory. Moreover, the distribution of efforts under medium control is hardly distinguishable between the two treatments (Appendix F.1 shows the cumulative distributions of effort). Overall, the descriptive evidence suggests that agents’ behavior differs in the two treatments mainly because positive reciprocity is stronger in the laboratory than online.

**Table 3:** Agents’ efforts as a function of the control level

	No control	Low control	Medium control
Laboratory	3.46	3.51	3.63
(1040 observations)	(2.73; 1; 2; 7)	(1.87; 2; 2; 5)	(1.21; 3; 3; 4)
Internet	2.79	3.15	3.60
(1160 observations)	(2.30; 1; 1; 5)	(1.61; 2; 2; 4)	(1.20; 3; 3; 4)

To complement the discussion on average behavior, we briefly report on the heterogeneity of agents’ reactions to (the absence of) control. About 29% (25%) of the online (laboratory) agents exert minimal effort at each control level in all 10 rounds, and about half of the agents do so in the majority of rounds (i.e. in at least 6 rounds). Control averse agents, i.e. agents who reduce their effort when controlled, are the next most frequent. Approximately 6% (10%) of agents in the Internet (laboratory) treatment express control aversion in all 10 rounds, and about 17% (28%) of the online (laboratory) agents are control averse in the majority of rounds. Agents who exert the same effort at each control level are relatively rare in both treatments. Only 3% of agents always react neutrally to control, about 10% do so in the majority of rounds, and nearly half of the agents adopt such a reaction in at least one round. Finally, in both treatments, positive reactions to control are very rare, they almost disappear over time, and hardly any agent reacts positively to control in all 10 rounds. More details on agents’ types are provided in Appendix F.3.^17^

### Testing Hypothesis 1: effort differences in the two treatments

To test formally whether differences in agents’ effort due to an increase in the level of control are larger online than in the laboratory, we rely on a series of regression models. The estimation method is linear mixed models where random intercepts at the agent and session levels are included. Random effects are assumed to be independent and to follow a normal distribution with mean zero. Thus, our regression models control for clustered errors at the agent and session levels by modelling explicitly the within-cluster error correlations.^18^ Models 1 to 3 predict effort differences under low and no control while models 4 to 6 predict effort differences under medium and low control. Table 4 summarizes the estimation results.

**Table 4:** Determinants of effort differences

	Dependent variable: Difference between effort under					
	Low and no control			Medium and low control		
	$e(2) - e(1)$			$e(3) - e(2)$		
Model	(1)	(2)	(3)	(4)	(5)	(6)
*Constant*	0.050	0.088	0.249	0.114	0.063	0.130
	(0.101)	(0.106)	(0.173)	(0.094)	(0.102)	(0.162)
*Int*	0.309**	0.286**	0.261*	0.332**	0.318**	0.298**
	(0.139)	(0.146)	(0.145)	(0.129)	(0.132)	(0.132)
*Half*2		− 0.062	− 0.062		− 0.045	− 0.045
		(0.061)	(0.061)		(0.051)	(0.051)
$Int*Half2$		0.047	0.047		0.046	0.047
		(0.084)	(0.084)		(0.069)	(0.069)
$b_A(2) - b_A(1)$		− 0.002	− 0.002			
		(0.002)	(0.002)			
$b_A(3) - b_A(2)$					0.001*	0.001*
					(0.001)	(0.001)
*Age*			− 0.135			− 0.039
			(0.139)			(0.128)
*Male*			− 0.025			0.084
			(0.141)			(0.129)
*Social*			− 0.092			0.015
			(0.178)			(0.164)
*Hum*			0.289			− 0.003
			(0.226)			(0.207)
*Tech*			− 0.330*			− 0.336**
			(0.183)			(0.168)
Observations	2200	2200	2200	2200	2200	2200
Log-likelihood	− 3349.874	− 3348.781	− 3344.465	− 2939.273	− 2937.337	− 2934.404

Standard errors in parentheses. ***(1%); **(5%); *(10%) significance level

In models 1 and 4, the effort difference is regressed against an intercept and the experimental condition where the dummy variable *Int* identifies the Internet treatment. Averaged over all agents and rounds, the effort difference is always positive but not significantly different from zero in the laboratory treatment and the difference always increases significantly in the Internet treatment. Regression results therefore show that differences in agents’ effort due to an increase in the level of control are significantly larger online than in the laboratory. Non-parametric tests based on session averages confirm that the two effort differences are significantly larger online than in the laboratory (Mann-Whitney tests; $p-values < 0.05$).

**Fig. 1:**
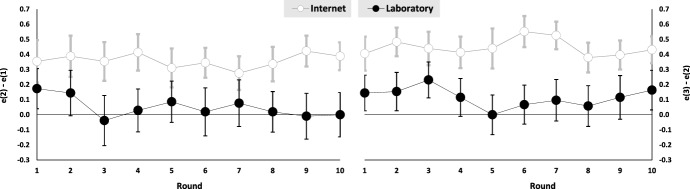
Effort differences over time (error bars represent standard errors)

The dynamics of effort differences indicate that the behavior of experienced agents is also supportive of our first hypothesis. Models 2 and 5 extend models 1 and 4 by including a dummy *Half2* for the second half of rounds (rounds 6–10), its interaction with the experimental condition Int*Half2, and a difference in beliefs [$b_A(2)-b_A(1)$ and $b_A(3)-b_A(2)$ respectively]. For both effort differences, the estimated coefficients of *Int* are significantly positive while those of *Half2* and Int*Half2 do not significantly differ from zero. Thus, effort differences and the influence of the distance in agency relationships are fairly stable over time.^19^ Figure 1 displays the effort differences over time.

The estimated coefficient of the belief difference is non-significantly different from zero for the categorical effect of control. For the marginal effect of control, on the other hand, the estimated coefficient is significantly positive. Though the estimated coefficient is very small, beliefs have a non-negligible impact on the effort difference as many agents strongly believe that the principal will enforce the medium level of control (as discussed at the end of this section, the average difference $b_A(3) - b_A(2)$ is about 50% in each treatment). Thus, despite the fact that agents react to the control level chosen by the principal, their beliefs impact their reactions to a marginal increase of control.

The estimated coefficients of models 2 and 5 barely change when demographic controls are included (age, gender and academic major)^20^ as shown in columns (3) and (6) of Table 4. Moreover, none of the demographics significantly impacts effort differences except for one of the fields of study.^21^

In sum, effort differences are positive in each treatment meaning that disciplining effects outweigh crowding effects of managerial control. Most importantly, the evidence supports our first hypothesis since effort differences are significantly larger online than in the laboratory. We now shed light on the mechanisms driving this finding.^22^

#### Testing Hypothesis 1a: reciprocity in the two treatments

The regression models on reciprocity are identical to those on effort differences except for the dependent variable. Models 1 to 3 predict reciprocity to low control, which corresponds to the difference between the effort under low control and the shifted effort under no control $(e(2) - max[e(1), 2])$, and models 4 to 6 predict reciprocity to medium control, which corresponds to the difference between the effort under medium control and the shifted effort under low control $(e(3) - max[e(2), 3])$. Table 5 reports our estimation results on reciprocity where more negative (sums of) coefficients indicate stronger reciprocity.

**Table 5:** Determinants of reciprocity

	Dependent variable: Reciprocity to					
	Low control			Medium control		
	$e(2) - max[e(1),2]$			$e(3) - max[e(2),3]$		
Model	(1)	(2)	(3)	(4)	(5)	(6)
*Constant*	− 0.435***	− 0.379***	− 0.247*	− 0.413***	− 0.359***	− 0.319***
	(0.078)	(0.083)	(0.132)	(0.068)	(0.077)	(0.120)
*Int*	0.261**	0.242**	0.222**	0.303***	0.284***	0.266***
	(0.107)	(0.113)	(0.111)	(0.093)	(0.098)	(0.098)
*Half*2		− 0.106*	− 0.106*		− 0.103**	− 0.103**
		(0.054)	(0.054)		(0.044)	(0.044)
$Int*Half2$		0.038	0.038		0.038	0.038
		(0.074)	(0.074)		(0.060)	(0.060)
$b_A(2) - b_A(1)$		− 0.001	− 0.001			
		(0.001)	(0.001)			
$b_A(3) - b_A(2)$					− 0.000	− 0.000
					(0.001)	(0.001)
*Age*			− 0.117			− 0.031
			(0.105)			(0.094)
*Male*			− 0.055			0.066
			(0.106)			(0.095)
*Social*			− 0.092			0.001
			(0.135)			(0.120)
*Hum*			0.331*			0.071
			(0.171)			(0.152)
*Tech*			− 0.220			− 0.239*
			(0.139)			(0.123)
Observations	2200	2200	2200	2200	2200	2200
Log-likelihood	− 3048.350	− 3045.536	− 3039.528	− 2603.731	− 2599.655	− 2596.600

Standard errors in parentheses. ***(1%); **(5)%; *(10%) significance level

**Fig. 2:**
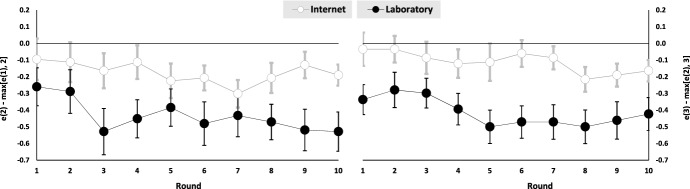
Reciprocity over time (error bars represent standard errors)

Averaged over all agents and rounds, reciprocity is always significant in the laboratory and significantly weaker online (models 1 and 4). Yet, online reciprocity to low (medium) control is significantly different from zero at the 5 (10) percent level (Wald tests: $p-value=0.018$ and $p-value=0.085$). Moreover, the results of models 2 and 5 show that experienced agents tend to be somewhat more reciprocal than inexperienced agents in both treatments. Figure 2 displays the extent of reciprocity over time. Also, the estimated coefficients of belief differences are never significantly different from zero. This findings indicates that agents’ beliefs do not significantly influence their reciprocal motives. Finally, the inclusion of demographic controls has little impact on the estimated coefficients of models 2 and 5 and none of the demographics consistently affects reciprocity as shown in columns (3) and (6) of Table 5.

In sum, reciprocal motives drive significantly agents’ behavior in both treatments, reciprocity tends to increase over time, and reciprocity is weaker online than in the laboratory which supports Hypothesis 1a.

#### Testing Hypothesis 1b: difference in efforts under no control in the two treatments

We now shed light on the nature of reciprocal motives in our two treatments by testing whether in the absence of control agents exert less effort online than in the laboratory. As before, we rely on linear mixed models with random intercepts for agents and sessions. Table 6 reports the estimation results.

**Table 6:** Agents’ effort in the absence of control

	Dependent variable: Effort under no control *e*(1)		
Model	(1)	(2)	(3)
*Constant*	3.462***	3.201***	2.873***
	(0.214)	(0.327)	(0.434)
*Int*	− 0.668**	− 0.606**	− 0.541*
	(0.295)	(0.294)	(0.292)
*Half*2		− 0.021	− 0.021
		(0.083)	(0.083)
$Int*Half2$		− 0.173	− 0.173
		(0.112)	(0.112)
$b_A(2)$		0.013***	0.013***
		(0.004)	(0.004)
$b_A(3)$		0.001	0.001
		(0.003)	(0.003)
*Age*			− 0.095
			(0.289)
*Male*			0.095
			(0.292)
*Social*			0.117
			(0.370)
*Hum*			0.157
			(0.469)
*Tech*			0.964**
			(0.380)
Observations	2200	2200	2200
Log-likelihood	− 4091.993	− 4077.437	− 4073.722

Standard errors in parentheses. ***(1%); **(5%); *(10%) significance level

Model 1 shows that, averaged over all rounds, the ‘no control’ effort exceeds the largest effort level enforceable by the principal in the laboratory and it is significantly lower online. Still, the average effort of online agents is significantly greater than the minimal effort level of 1 (based on a two-sided Wald test, we reject the null hypothesis that the two coefficients sum to one: $p-value < 0.01$). According to model 2, the extent to which the agent’s effort under no control differs between the two treatments is fairly stable over time and the impact of beliefs is either insignificant or small [indeed, as indicated in the next part of the section, the average $b_A(2)$ is less than 20% in each treatment]. The estimated coefficients of model 2 are hardly affected when including demographic controls as shown by model 3. Except for one of the fields of study none of the demographics significantly impacts the agent’s effort under no control.^23^

Our regression results support Hypothesis 1b. Overall, our supporting evidence for hypotheses 1a and 1b strongly suggests that agents’ behavior is partly driven by positive reciprocity in our two treatments. Accordingly, the effectiveness of control is undermined by the presence of hidden benefits of abstaining from control which are stronger in the laboratory than in the Internet treatment.

#### Agents’ beliefs

Averaged over all agents and rounds, expected frequencies of no control, low and medium control are 14%, 19% and 67% (12%, 17% and 71%) in the Internet (laboratory) treatment. We fail to reject the null hypothesis that the distribution of individual beliefs, averaged across rounds for each agent, about the frequency of principals who choose the no, low or medium control level is the same in the two treatments (Wilcoxon rank-sum test: $p-values > 0.1$).^24^ To assess the correctness of agents’ beliefs, we compute mean squared differences between beliefs and actual frequencies of control levels and we average them across rounds for each agent. When considering all or the second half of rounds, we never reject the null hypothesis that the distribution of the mean squared difference is the same in the two treatments (Wilcoxon rank-sum test: $p-values > 0.1$). Experienced agents do not predict principals’ control decisions significantly better in any of the two treatments. Further details about agents’ beliefs are given in Appendix F.4.

### Testing Hypothesis 2: levels of control in the two treatments

Overall frequencies of control levels are very similar in the two treatments. Averaged over all principals and rounds, frequencies of no control, low and medium control are 15%, 15% and 70% (15%, 18% and 67%) in the Internet (laboratory) treatment. As the session progresses, the medium (low) control level is chosen somewhat more often (less often). In the second half of rounds, frequencies of no control, low and medium control are 13%, 12% and 75% (11%, 18% and 71%) in the Internet (laboratory) treatment. Principals who choose medium control represent the most frequent and most stable type. In the Internet (laboratory), 26% (28%) always choose medium control, and 72% in both treatments do so in the majority of rounds. Principals who consistently choose no or low control hardly exist.

We test our second hypothesis with the help of linear mixed models where the dependent variable is the level of control implemented by the principal and random intercepts at the principal and session levels are included. Table 7 reports the estimation results.^25^ According to model 1, averaged over all principals and rounds, the estimated control level is not significantly different in the two treatments. Moreover, the results of model 2 show that the extent of managerial control increases over time. Model 3 extends model 2 by including differences in beliefs and their interactions with the treatment dummy. The higher the effort increase principals expect when imposing low rather than no control the higher the control level they impose [the coefficient of $b_P(2) - b_P(1)$ is significantly positive]. Likewise, the higher the effort increase principals expect when imposing medium rather than low control the higher the control level they impose [the coefficient of $b_P(3) - b_P(2)$ is significantly positive]. The beliefs of principals impact their control decisions similarly in the first and in the second half of rounds and the magnitude of the impact is very similar in the two treatments (regression results of model 4). Finally, the regression results are robust to the inclusion of demographic controls and none of the demographics significantly impacts the extent of managerial control (regression results of model 5).

To summarize, the regression results do not support our second hypothesis since the estimated control level is not significantly different in the two treatments.^26^

**Table 7:** Determinants of the control intensity

	Dependent variable: Level of control				
Model	(1)	(2)	(3)	(4)	(5)
*Constant*	2.528***	2.452***	2.321***	2.332***	2.301***
	(0.049)	(0.052)	(0.047)	(0.048)	(0.077)
*Int*	0.014	0.010	− 0.015	− 0.016	− 0.022
	(0.067)	(0.071)	(0.065)	(0.066)	(0.067)
*Half*2		0.152*** (0.036)	0.122*** (0.035)	0.092** (0.041)	0.093** (0.041)
$Int*Half2$		0.008 (0.049)	0.003 (0.048)	− 0.013 (0.061)	− 0.013 (0.061)
$b_P(2) - b_P(1)$			0.134*** (0.020)	0.123*** (0.025)	0.123*** (0.025)
$b_P(3) - b_P(2)$			0.135*** (0.022)	0.124*** (0.026)	0.123*** (0.026)
$Int*[b_P(2) - b_P(1)]$			0.022 (0.027)	0.024 (0.032)	0.024 (0.032)
$Int*[b_P(3) - b_P(2)]$			− 0.010 (0.028)	− 0.007 (0.034)	− 0.007 (0.034)
$Half2*[b_P(2) - b_P(1)]$				0.026 (0.036)	0.025 (0.036)
$Half2*[b_P(3) - b_P(2)]$				0.027 (0.037)	0.026 (0.037)
$Int*Half2*[b_P(2) - b_P(1)]$				0.010 (0.051)	0.010 (0.051)
$Int*Half2*[b_P(3) - b_P(2)]$				0.006 (0.051)	0.006 (0.051)
*Age*					0.030 (0.060)
*Male*					0.096 (0.063)
*Social*					− 0.045 (0.074)
*Hum*					− 0.018 (0.083)
*Tech*					− 0.055 (0.089)
Observations	2200	2200	2200	2200	2200
Log-likelihood	− 2145.224	− 2125.083	− 2018.572	− 2016.659	− 2014.685

Standard errors in parentheses. ***(1%); **(5%); *(10%) significance level

#### Principals’ beliefs

Principals correctly expect agents to exert more than the minimal effort under no control and they also correctly expect efforts to increase with control. Averaged over all principals and rounds, expected efforts under no, low and medium control are 2.58, 3.18 and 3.82 (2.93, 3.46 and 4.02) in the Internet (laboratory) treatment. Expected effort, averaged across rounds for each principal, significantly increases with the level of control in each treatment (Wilcoxon matched-pairs signed-ranks tests: $p-value < 0.01$ in all four cases). Since agents’ effort differences are significantly different from zero only in the Internet treatment, principals seem unaware that the distance in agency relationships influences how agents’ efforts vary with the level of control. In a similar vein, Dickinson and Villeval (2008) conclude that the nature of the agency relationship does not significantly affect the monitoring of their principals.

**Fig. 3:**
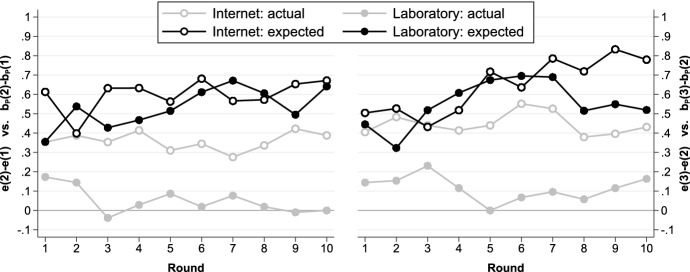
Effort differences expected by principals versus actual effort differences over time

Figure 3 contrasts the average effort differences expected by principals (black lines) with the actual effort differences (grey lines) over time. Principals expect effort differences to be clearly positive in every round even though actual average effort differences are often close to zero in the laboratory. The fact that principals’ beliefs do not adjust in the right direction is hardly surprising since they only observe the agent’s effort for the control level they have chosen. In particular, a principal who always chooses a medium control level will never learn that some agents exert more effort in case of no control. Monetary payoff-maximizing principals should always enforce medium control (see Fig. 1) and on average principals correctly believe that enforcing medium control is the decision which maximizes their monetary payoffs. Further details about principals’ beliefs and monetary payoffs are given in Appendices F.6, F.7 and F.8.

## Conclusion

There is by now conclusive evidence that the effectiveness of control is undermined by agents’ reciprocal behavior in a laboratory principal-agent game where the principal can control the agent by implementing a minimum effort requirement before the agent chooses an effort costly to her but beneficial to the principal. We report a first attempt at exploring experimentally the influence of workplace arrangements on the effectiveness of control by comparing the online and laboratory behavior in a straightforward extension of the principal-agent game. Our design captures meaningful differences between working from home and working at the office arrangements as online subjects enjoy greater anonymity than lab subjects, they interact in a less constrained environment than the laboratory, and there is a larger physically-oriented social distance between them. We find that control is significantly more effective in distant than in close agency relationships even for experienced agents. Reciprocal motives drive agents’ behavior in both treatments, but reciprocity is significantly weaker online than in the laboratory. Additionally, in the absence of control, agents exert significantly less effort online than in the laboratory. In line with Burdin et al. (2018), agents’ reactions to (the absence of) control suggest that the effectiveness of control is undermined by the presence of hidden benefits of abstaining from control which are stronger in the laboratory than in the Internet treatment. We also observe that in both treatments principals often choose the highest control level which maximizes their monetary payoffs.

Allowing employees to work from home is an important form of granting them autonomy. Recent evidence indicates that, contrary to other alternative work arrangements, employees value highly WFH arrangements and that they would be willing to take lower wages for the ability to work from home (Mas and Pallais 2017). Our results suggest that WFH arrangements have additional benefits for employers as the imposition of tougher controlling on employees who work from home is less likely to crowd out their motivation. On the other hand, working in the office benefits from positive reciprocity in closer personal relationships as an additional source of motivation.

Clearly, this evidence should be viewed as a first step in understanding the influence of workplace arrangements on the reactions to (the absence of) control. Given its practical relevance for organizations, we hope that future experimental work will build upon our weak manipulation to further characterize the effectiveness of control strategies in different workplace arrangements.

## Electronic supplementary material

Below is the link to the electronic supplementary material.

Supplementary material 1 (pdf 5218 KB)

## Footnotes

1 Even before the pandemic struck, remote work was accelerating in the U.S. Indeed, recent data from the American Time Use Survey show that the share of U.S. workers doing some or all of their work at home grew from 19 percent in 2003 to 23 percent in 2017 (the American Time Use Survey, which is conducted by the U.S. Census Bureau for the Bureau of Labor Statistics, is a continuous survey about how individuals age 15 and over spend their time). A similar trend is observed in Europe with almost a third of the employees carrying out their work in multiple locations in 2017 (Eurofound 2017).

2 In the principal-agent game the agent’s effort corresponds to a monetary cost rather than a real effort. There is however supporting evidence that monetary costs capture well the mental or physical costs in a real effort task (Dutcher et al. 2015; Riener and Wiederhold 2016).

3 Additionally, lab subjects might feel scrutinized by the experimenter which could lead lab agents to react more negatively to managerial control than online agents. However, we believe that our experimental procedures induced a rather comparable level of experimenter-subject anonymity in both treatments especially as subjects always collected their earnings in the laboratory. Note also that Barmettler et al. (2012) fail to confirm that the absence of experimenter-subject anonymity in laboratory experiments leads to an overestimation of the importance of social preferences.

4 Still, as is common practice in economic experiments, lab subjects were seated in private cubicles, they gave responses anonymously and they were paid in private.

5 The hidden benefit of abstaining from control is the expected gain in earnings a principal enjoys when not controlling an agent who is intrinsically motivated to act in the principal’s interest and exerts more effort in the absence of control than under control.

6 Alternatively, Kessler and Leider (2016) investigate whether the fairness of the process by which control is imposed affects whether or not control will backfire. They find that control leads agents to withhold effort only when procedural fairness concerns are ignored and control is imposed unilaterally with an asymmetric effect on the agent.

7 To avoid demand effects, we did not distinguish in the instructions and on the decision screens between “no control” and a level of control which restricts the agent’s choice set. All three control levels were phrased in the same way, namely that the principal forces the agent to exert an effort level of at least 1, 2 or 3. Thus, our instructions are conservative with respect to the categorical effect of control. See the experimental screens in Appendix D.1.

8 Concretely, assume that 12 agents and 12 principals take part in the session. In the first repetition, agent 1 interacts with principal 1, agent 2 interacts with principal 2, ..., and agent 12 interacts with principal 12. In the second repetition, agent 1 interacts with principal 2, agent 2 interacts with principal 3, ..., and agent 12 interacts with principal 1. And in the last repetition, agent 1 interacts with principal 10, agent 2 interacts with principal 11, ..., and agent 12 interacts with principal 9. Full details about our matching protocol are available in Appendix D.2.

9 Experimental economists have expressed the concern that subjects’ behavior may differ when beliefs are elicited as compared to when they are not (Schotter and Trevino, 2014 as well as Schlag et al. 2015 extensively discuss this topic). Two reasons can be advanced as to why this is not a serious concern in our experiment. First, our payment scheme eliminates the hedging incentives, i.e., high earnings in the belief elicitation task cannot compensate for low earnings in the game, or vice versa. Indeed, each subject is paid either for the accuracy of her guesses or she receives the payoff associated with the game outcome. Second, economic experiments do not agree on whether and how belief elicitation affects other-regarding considerations in social dilemmas. There is therefore no conclusive evidence to support the claim that belief elicitation biases subjects’ propensity to reciprocate in our experiment.

10 The first laboratory sessions took place in the experimental laboratory of the Max Planck Institute of Economics in Jena while the next laboratory sessions took place in *Lakelab*, the laboratory for economic experiments in Konstanz. Both laboratories strictly adhere to a non-deception policy.

11 We do not elaborate on the survey as it is not central to the study at hand.

12 Online subjects collected their earnings about three days after the session, once the official lottery draw had determined their payoffs. In contrast, laboratory subjects were paid immediately after the session as usual. Monetary incentives might therefore be less salient in the online than in the laboratory treatment. Former experimental evidence suggests that Frey’s hypothesis is less likely to be validated because of this procedural difference. Indeed, Ziegelmeyer et al. (2012) report an extension of FK’s low and medium control treatments where both principals and agents are paid according to a flat participation fee and they observe that control is less effective under hypothetical than under real incentives.

13 At the time of the experiment, the purchasing power index of Thuringia (federal state of Jena, East Germany) was about 84 whereas the purchasing power index of Baden-Württemberg (federal state of Konstanz, West Germany) amounted to 107 (GfK 2014).

14 In one laboratory session in Jena, two participants were prevented from completing the session because of a network connection failure. In the online treatment, three subjects in Konstanz dropped out due to technical issues, and no subject dropped out intentionally. For each dropout, one participant of the other role was excluded from further participation. Participants who dropped out or were excluded were informed privately on their screens while the session proceeded smoothly for the remaining participants. We restrict our data analysis to participants who completed the experiment.

15 The hidden cost of control is the expected loss in earnings a principal suffers from controlling an agent who is intrinsically motivated to act in the principal’s interest and exerts less effort under control than in the absence of control.

16 If the principal cares solely about his monetary payoffs then he should limit the agent’s effort discretion to the largest possible extent. The fact that the variability in efforts reduces as the level of control increases provides another rationale for principals to select the medium control level.

17 We estimated three simple linear models with random effects at the agent level to check whether the principal’s choice in the previous round affects the effort under no, low and medium control. We find that the principal’s choice in the previous round never significantly affects the agent’s effort. Details are available from the authors upon request.

18 A more robust approach uses clustering at the session level in the computation of the variance-covariance matrix. However, these cluster-robust standard errors require a large number of clusters and we conducted only sixteen sessions. Moreover, we observe that, for all regression models, the estimate of the random intercept at the session level is basically zero. Indeed, the nature of our matching protocol limits the degree to which the behavior of agents in the same session is correlated. Running the same regressions without a random intercept at the session level generates almost identical standard errors. Using OLS regressions with clustering at the agent level also gives similar results.

19 Linear combinations of the coefficients *Int* and $Int*Half2$ are significantly different from zero, confirming that in the second half, effort differences are also larger online than in the laboratory (Wald tests: $p-values < 0.05$).

20 *Age* equals 1 if the subject’s age is at the median or above and 0 otherwise. *Social* equals 1 if the field of study belongs to the category “behavioral & social sciences except economics” and 0 otherwise. *Hum* equals 1 if the field of study belongs to “humanities” and 0 otherwise. *Tech* equals 1 if the field of study belongs to “engineering, life & natural sciences” and 0 otherwise.

21 The significant impact of *Tech* on effort differences is not robust when interacting the field of study with the experimental condition. Note also that our demographic controls affect the impact of the experimental condition on the difference between effort under low and no control in the first half of rounds where the estimated coefficient of *Int* is only weakly significant in model 3 ($p-value=0.070$). The linear combination of the coefficients *Int* and $Int*Half2$ differs from zero at the 5 percent level (Wald test: $p-value=0.033$) meaning that the effort difference $(e(2)-e(1))$ is still significantly larger online than in the laboratory in the second half of the session.

22 Since differences in effort levels are not linear with respect to the agent’s effort costs, we performed robustness checks with differences in effort costs as the dependent variable. Additionally, we included in models 3 and 6 the level of control chosen by the principal in the previous round since the feedback received by agents could influence their behavior and generate dynamic session-effects (Fréchette 2012). The results of these robustness checks are in line with the regression results reported in the main text.

23 Our demographic controls affect the impact of the experimental condition on efforts under no control in the first half of rounds where the estimated coefficient of *Int* is only weakly significant in model 3 ($p-value=0.064$). The linear combination of *Int* and $Int*Half2$ differs from zero at the 5 percent level (Wald test: $p-value=0.015$) meaning that efforts under no control are significantly smaller online than in the laboratory for the second half of rounds.

24 Differences between agents’ beliefs in the two treatments are also non-significant in the second half of rounds (Wilcoxon rank-sum test: $p-values > 0.1$) and agents expect slightly higher effort control over time. In the second half of rounds, expected frequencies of no control, low and medium control are 13%, 16% and 71% (11%, 14% and 75%) in the Internet (laboratory) treatment.

25 As for the analysis of agents’ effort decisions, the estimate of the random intercept at the session level is basically zero for all regression models of the principals’ control decisions and the same regressions without a random intercept at the session level generate almost identical standard errors. Using OLS regressions with standard errors clustered for subjects also gives similar results.

26 Linear mixed models implicitly assume that principals’ choices are measured on a continuous scale. However, principals choose their levels of control from three ordered categories (no control, low control, and medium control). We checked the robustness of our results by estimating regression models that account for the ordinal nature of the dependent variable, namely ordered probit models. We report the regression results in Appendix F.5 of the supplementary material. These regression results confirm the main text findings: (1) Principals do not enforce more effort on the internet than in the laboratory; (2) Experienced principals control more; and (3) The more principals expect an increase in agents’ effort due to an increase in the level of control, the higher the control level they choose. We report the results of linear mixed models in the main text for the ease of interpretation.
