# Supplementary material for: Reactions to (the absence of) control and workplace arrangements: experimental evidence from the internet and the laboratory
Source: Exp Econ. 2020 Aug 13;23(4):933–60. doi: 10.1007/s10683-020-09666-8 (PMC7424963; doi:10.1007/s10683-020-09666-8)
Supplement: Supplementary file 1 — Supplementary material 1 (pdf 5218 KB) [file 10683_2020_9666_MOESM1_ESM.pdf]

Supplementary material for

Reactions to (the Absence of) Control and Workplace Arrangements: Experimental Evidence from the Internet and the Laboratory

[For Online Publication]

In Appendix A, Table 1 (respectively Table 2) reports the common (respectively distinct) characteristics of the two treatments. Appendix B details the participation process of our study, from registration to payment. Appendix C presents the participation rates over the course of the study. Appendix D shows the main screens of the study (translated to English) and explains how technical issues specific to online sessions were dealt with. In Appendix E we compare the Jena and Konstanz data. Appendix F reports statistical analyses that complement those of the main text. Finally, Appendix G compares the quality of our laboratory and online data.

A Common and distinct characteristics of the two treatments

**General features**	
Subject pool	Students
Software	Online platform
Registration for the study	ORSEE invitation email with a link to the online platform<br>For registration subjects enter their gender, month and year of birth, nationality, mother tongue, and email address
**Survey**	
Login	Survey token sent via email; login requires authentication
Location	Completed at the subjects' place of choice
Time	Time frame of a few days; time gap before the experiment
Content	Sociodemographics, attitudes towards trust and control, work experience
**Experiment**	
Registration for session	At the end of the survey
Time of the session	Prearranged start time in the afternoon or evening
Login	Session token sent via email one to two hours before session start; login page available 15 minutes before session start; login requires authentication
Experimental screens	1. Countdown screen before start of the session<br>2. Introductory screens<br>3. Instructions<br>4. Control questions<br>5. Beliefs and choices in 10 rounds<br>6. Feedback about the partner's choice after each round<br>7. Details about the lottery procedure<br>8. Information about payment<br>9. Feedback about correctness of beliefs
Payment	Cash in the laboratory (show-up fee + survey's flat payment + payment of a choice or a belief depending on the outcome of the lottery draw)

Table 1: Common characteristics of the two treatments.

	Laboratory	Internet
Location of the experiment	Laboratory	Place of choice
Random draw to determine payoff-relevant choices/beliefs	A randomly selected subject draws the lottery numbers	Official lottery draw on a specified day in the future
Timing of payment	At the end of the session	A few days after the session

Table 2: Distinct characteristics of the two treatments.

B The participation process (from registration to payment)

The study was conducted on an internet platform developed by the authors. The platform facilitates the conduct of interactive online experiments since it allows all subjects of a given session to interact in real time and it takes care of technical issues specific to online sessions (see Appendix D.2 for details). Participants only need a web browser in which javascript is enabled. Figure 1 illustrates the participation process of our study. Unless stated differently, this process was identical for the laboratory and the Internet treatments.

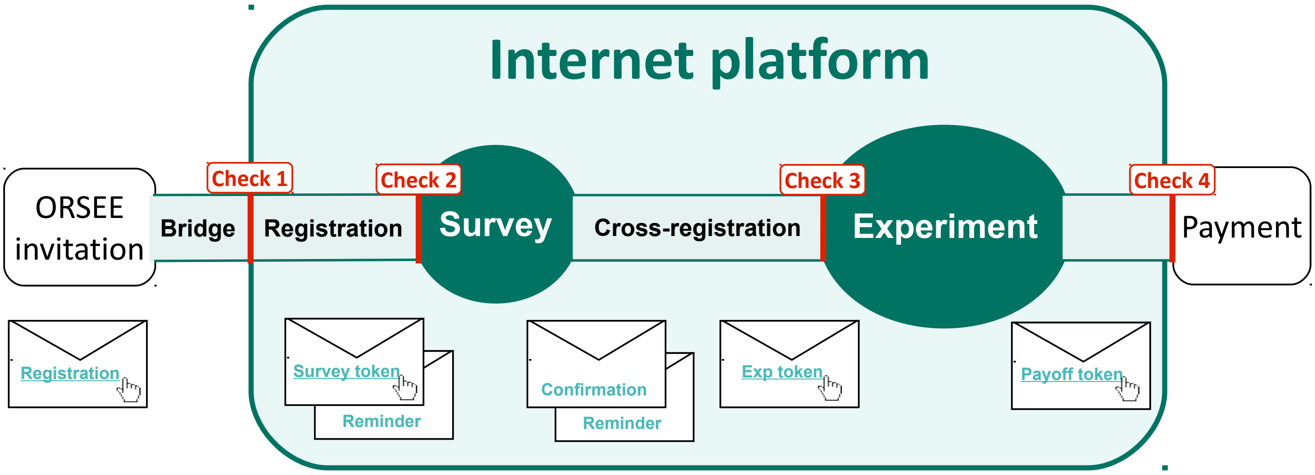

Figure 1: The participation process.

Notes: Red markers indicate the four checkpoints where the eligibility of participants was verified.
The envelopes symbolize emails sent to participants.

Participants received an invitation email via ORSEE with a link to the registration pages of the study (see translated screens in Appendix D.1). To register for the study (check 1), students had to enter their gender, month and year of birth, nationality, mother tongue and email address. A bridge from the internet platform to ORSEE allowed us to verify a participants' eligibility. Concretely, registration was successful only if both the email address and gender entered on the registration screen matched the email address and gender of a subject invited via ORSEE.

Registered participants immediately received an email with a token (a personalized link) to the survey. The survey token lead to the login page of the survey (check 2) where participants were asked to type in their email address and the last digits of their student number. Eligible participants were then introduced to the study and they answered the survey questions. Up to two reminders were sent to registered participants who had not yet completed the survey. At the end of the survey participants could register for an experimental session. This cross-registration was implemented such that survey data could easily be linked to experimental data.

Once registered for the experiment, participants received a confirmation email with the date and time of the experimental session for which they had registered. In addition, participants received reminder emails 72 (if applicable) and 24 hours before the starting time of their experimental session. As we were concerned that some participants might forward their link to another person, session tokens to the experiment were

sent only one or two hours before an experimental session started. This token lead to the login page of the experiment (check 3) where participants had to enter their email address and the last digits of their identification card. Participants were aware that their eligibility would be checked but, to prevent participants from exchanging tokens, they did not know in advance which information would be required to login. Participants who entered the experiment in time were able to participate.

Participants who completed an experimental session as well as participants who dropped out for external reasons were eligible for payment. In the Internet treatment, the choice or belief that was selected for payment was determined at random according to the German official lottery numbers. In the laboratory treatment, the payoff relevant choice or belief was determined at random according to lottery numbers drawn by a randomly selected subject at the end of the experimental session. Once payoffs had been computed based on the lottery numbers, participants received an email with a token to a page showing their final payoff. When they were informed of their final payoffs, participants also learned about the correctness of their beliefs. Finally, in order to receive their payment (check 4), participants had to name their email address and present their student ID as well as their identity card to verify the information entered at previous checkpoints.

C Participation rates over the course of the study

Table 3 shows the participation rates for our laboratory and Internet treatments over the course of the study. For each treatment and for each stage of the study, the table provides the number of subjects that are involved (columns "N"), the share of subjects involved in the current stage out of those involved in the previous stage (columns "% previous row"), and the share of subjects involved in the current stage out of those who registered for the survey (columns "% total"). Overall, the participation rates are rather high and largely similar in both treatments.

	Laboratory			Internet		
	N	% previous row	% total	N	% previous row	% total
Registered for the survey	245		100	298		100
Survey started	237	97	97	283	95	95
Survey completed	237	100	97	283	100	95
Registered for the experiment	232	98	95	280	99	94
Experiment started	212	91	87	258	92	86
Instructions completed	212	100	87	247	96	83
Control questions completed	212	100	87	247	100	83
Round 1 started	212	100	87	241	98	81
Round 1 completed	212	100	87	238	99	80
Round 5 completed	210	99	86	232	97	78
Round 10 completed	208	99	85	232	100	78

Table 3: Participation rates in the laboratory and on the Internet.

To invite subjects, we created two separate experiments in ORSEE for the laboratory and Internet treatments. As we expected more dropouts in the interactive part of the Internet treatment and no dropouts in the interactive part taking place in the laboratory, we started out with a higher number of subjects for the Internet treatment. Thus, slightly more than half of the subjects were randomly assigned to the Internet experiment and slightly less than half of the subjects were randomly assigned to the laboratory experiment in ORSEE. For technical reasons, surveys on our online platform require a predefined number of subjects. Again, because we expected dropouts during the interactive part online, we set a higher limit of subjects for the online surveys than for the laboratory surveys (in total, 298 vs. 245 subjects). In both treatments, the maximal number of subjects registered for the survey.

We now explain the participation rates over the course of the study, referring to the columns "% previous row" of Table 3. Out of the subjects who registered for the survey, 97% (95%) in the laboratory (Internet) treatment started with the survey (i.e., they answered the first survey question) and all of them also completed the survey (i.e., they answered the last survey question). Thus, there were no dropouts

during the survey. 98% (99%) of the laboratory (Internet) subjects who completed the survey registered for an experimental session at the end of the survey.

91% (92%) of the laboratory (Internet) subjects who registered for an experimental session also started with the experiment. 100% (96%) of the laboratory (Internet) subjects who started with the experiment read the three pages of the instructions and arrived at the control questions.[1] All subjects who started with the control questions also completed them in both treatments. At this stage, in some Internet sessions, a randomly selected subject had to be excluded because an odd number of subjects was left after the control questions. (In the laboratory sessions, only an even number of subjects were admitted to enter the laboratory.) 100% (98%) of the laboratory (Internet) subjects who completed the control questions started round 1 (i.e., they entered their beliefs). 100% (99%) of the laboratory (Internet) subjects who started round 1 also completed round 1 (i.e., they made their choices). 99% (97%) of the laboratory (Internet) subjects who completed round 1 also completed round 5, and 99% (100%) of the laboratory (Internet) subjects who completed round 5 also completed round 10.[2] These are the 208 (232) subjects of the laboratory (Internet) treatment our paper is based on.

D Screens of the study and trouble handling

This Appendix contains translated screens of the study and explanations on trouble handling. The registration screens and the most important experimental screens (instructions, control questions, belief elicitation and decision screens, feedback screens after each round) are provided in Appendix D.1. Trouble handling is explained in Appendix D.2.

On the internet, limited data quality due to an inevitable loss of control compared to the laboratory is of a major concern. To prevent participants from just clicking through the pages, we developed a read mode and an edit mode. In both treatments, screens appeared in the read mode first where all active items (buttons, input elements) were locked. Only after a few seconds as defined by the experimenter for every screens, the items became unlocked for participants to continue.

A major challenge in interactive real-time experiments on the internet is to synchronize participants without having reliable information about their physical presence as in the laboratory. We approached this problem by organizing an experimental session in blocks (instructions, control questions, each round). Each block had a maximum timeout and participants were informed of the timeout before they entered a block.[3] A common start of a session and of blocks within a session was ensured by countdown screens before each block. Countdown screens redirected participants automatically to the next page. Participants who completed a block before the timeout were redirected to a waiting screen. Participants who failed to complete a block within the timeout were excluded as detailed in Appendix D.2. The session continued once all participants completed a block before the timeout, or once the timeout was reached.

D.1 Screens of the study

Most screens are identical for both treatments. In particular, exactly the same instructions, belief elicitation screens and decision screens were always used. Merely the registration and payment screens were slightly adjusted to account for the environment of the experimental session (place of choice or laboratory) and the lottery procedure (official lottery numbers or lottery numbers drawn by a participant). The screens provided here refer to the internet experiment. The instructions were identical for principals (participant A) and agents (participant B).

[1]At the instructions stage, we had a few dropouts in our first two Internet sessions because some subjects expected to proceed automatically and missed the "Continue" button at the end of the instructions. Thus, they exceeded the time limit for the instructions and were excluded. We fixed this issue after the first two sessions by adding a warning message which popped up shortly before timeout. The first laboratory session was conducted after this problem had been resolved, and therefore, it never occured in the laboratory.

[2]We had four dropouts in the interactive part of a laboratory session. Two subjects in two different rounds were stuck on a screen because of a server problem. Consequentially, their partners had to be excluded from further participation as well. (The procedure was explained to the subjects who dropped out on their screens, while the experiment proceeded smoothly for the remaining subjects in that session.)

[3]To account for learning, time limits were higher in the first round than in later rounds.

One case out of three

"One case out of three" is a study by the Max Planck Institute of Economics in Jena. We are part of the Max Planck Society for the Advancement of Science, an independent and non-profit research organization. The Max Planck Society takes up innovative research areas and enjoys worldwide recognition for excellent basic research.

We regularly conduct interactive experiments in our computer laboratory in Jena. In these experiments, students can earn money based on their decisions. The participants of this internet study are also students. The same rules apply as for our laboratory experiments. According to our strict rules of authenticity, we do not provide erroneous information intentionally.

What is this study about?

"One case out of three" is an economic study in the field of motivation research. It consists of two parts that take place on different days. The first part contains a few **personal questions**. The second part is an **interactive experiment**.

How can I earn money?

You will be paid for your participation in this study. For answering the personal questions you will receive a fixed amount. Your payment for the experiment will depend on your decisions, as well as on the decisions of the other participants. You can only be paid for your participation if you complete both parts.

How long does the study take?

You will need **about 10 minutes** to complete the **survey**. The **experiment** will take **about 90 minutes**. You will find the respective dates on the next page.

Go to registration

One case out of three

It is up to you when you complete the **survey**. At the end of the survey you can register for the **experiment**. The **payment** will take place in our computer laboratory in the Goethe-Galerie.

Survey	Experiment	Payment
Till Friday, 11/19/2010, 11:55 PM	Monday, 11/22/2010, 6:00-7:30 PM<br>Tuesday, 11/23/2010, 6:00-7:30 PM	Thursday, 11/25/2010, 2:00-8:00 PM

At the beginning of the survey and of the experiment as well as for payment, your eligibility will be checked. We appreciate your understanding in this matter. Your answers in this study will not be connected to you as an individual. Your data will be treated as **strictly confidential** and will only be used for scientific purposes. If you want to register for this study, please provide us with the following data:

Gender ○ female ○ male

Date of birth [ ] 19 [ ]

Citizenship [ ]

Mother tongue [ ]

Email* [ ]

** Your email address at which we have contacted you.*

If you have questions, please visit our FAQs or contact us at internetexperiment@econ.mpg.de.

In this experiment, you will make decisions for which you will receive points. Remember, the exchange rate from points to euros is as follows:

1 point = 0.15 euros

General procedure

One half of the participants will be named A and the other half of the participants will be named B. The experiment consists of **10 rounds**. You will learn whether you are participant A or B before the beginning of the first round. You will keep your name over all rounds.

In every round, for all participants, one participant A and one participant B will be matched **randomly**. The participant you are matched with in a given round will never be matched with you in any later round. No participant will learn with whom he has interacted.

Every round follows the same procedure. Participants make **decisions** in the following situation: Imagine an arrangement where participant B completes a task for participant A. Participant **B decides** on a symbolic effort level (at least 1 and at most 10) to complete the task. The effort of B is beneficial for A and costly for B. Accordingly, the higher the effort level of B, the higher the income of A and the lower the income of B. Participant **A decides** whether to force participant B to exert a minimum effort level of 1, 2, or 3.

Before decisions are made in a given round, all participants **guess** what other participants will decide in that round.

On the next pages, you will learn in more detail about the decisions, the guesses, and the incomes.

Next, we explain the decisions.

Page ▷
1 Next

The decisions

Both participants make their decisions at the same time. Therefore, each participant makes his decision **without knowing** what the other participant decides.

Participant A decides on **one** of the following three options:

- **Option 1**: A forces B to exert an effort level of **at least 1**.
- **Option 2**: A forces B to exert an effort level of **at least 2**.
- **Option 3**: A forces B to exert an effort level of **at least 3**.

Participant B decides on an effort level for **each** of the following three cases:

- **Case 1**: A chooses option 1. Thus, B can choose any effort level **between 1 and 10**.
- **Case 2**: A chooses option 2. Thus, B can choose any effort level **between 2 and 10**.
- **Case 3**: A chooses option 3. Thus, B can choose any effort level **between 3 and 10**.

Consequently, participant A makes **one** decision and participant B makes **three** decisions. The option chosen by A determines the case in which the effort level chosen by B is relevant for calculating the incomes of both participants. The following table shows the incomes of both participants depending on the effort level of B.

**Effort level of B**	**1**	**2**	**3**	**4**	**5**	**6**	**7**	**8**	**9**	**10**
Income of A (in points)	1	16	29	41	53	64	75	82	87	90
Income of B (in points)	99	98	96	93	89	83	75	65	51	35

Next, we explain the guesses and the incomes.

◁ Page ▷
Previous 1 2 Next

The experiment: instructions

The guesses

Participant A guesses the effort level all participants B will choose on average in case 1, in case 2, and in case 3. **Participant B** guesses how many participants A will decide on option 1, on option 2, and on option 3. Thus, each participant makes three guesses per round. The guesses always refer to the current round. If your guess is close to the actual value then you will receive **70 points**. Otherwise you will receive **20 points**.

End of the rounds

At the end of each round, you will learn the decision of the participant you were matched with in that round. You will also learn what your incomes resulting from your decisions are. Only after the experiment is over will you find out whether your guesses were right.

Summary and calculation of the payment

The experiment consists of 10 rounds. In every round, for all participants, one participant A and one participant B will be matched. A decides on the minimum effort level he enforces from B. B decides on an effort level for each potential decision of A. Before the decisions are made, each participant guesses what the other participants will decide.

After the experiment is over, **one** round will be selected at random for payment. Each round has the same probability to be selected. Whether the payment is calculated based on the decisions **or** on the guesses of that round will again be selected at random and with equal probability. Only one of the three guesses will be selected and each guess is given the same probability.

Click "Next" when you have finished reading the instructions.

◁ Page ▷
Previous 1 2 **3** Next

The experiment: control questions

INSTRUCTIONS

To answer the control questions please see the respective incomes in the table.

**Effort level of B**	**1**	**2**	**3**	**4**	**5**	**6**	**7**	**8**	**9**	**10**
Income of A (in points)	1	16	29	41	53	64	75	82	87	90
Income of B (in points)	99	98	96	93	89	83	75	65	51	35

Question 1: Suppose participant A forces B to exert an effort level of at least 1. Participant B chooses an effort level of 1 in case 1, of 2 in case 2, and of 3 in case 3.

What are the incomes?

Income of A: ____ points

Income of B: ____ points

Question 2: Suppose participant A forces B to exert an effort level of at least 2. Participant B chooces an effort level of 7 in case 1, of 7 in case 2, and of 7 in case 3.

What are the incomes?

Income of A: ____ points

Income of B: ____ points

Question 3: Suppose participant A forces B to exert an effort level of at least 3. Participant B chooces an effort level of 6 in case 1, of 5 in case 2, and of 4 in case 3.

What are the incomes?

Income of A: ____ points

Income of B: ____ points

Please answer the questions.

The experiment: make a guess!

INSTRUCTIONS

What do you guess is the average effort level of **all** participants B in case 1, in case 2, and in case 3 in this round? If your guess does not differ by more than 0.5 from the actual average effort level for a given case then you will receive **70 points**. Otherwise you will receive 20 points.

No other participant will ever know your answer. Please always insert an integer without decimals.

I guess the average effort level of all participants B is ...

Case 1: about [ ] when they are **forced** to exert an effort level of **at least 1**.

Case 2: about [ ] when they are **forced** to exert an effort level of **at least 2**.

Case 3: about [ ] when they are **forced** to exert an effort level of **at least 3**.

The general income table is shown once more for visualization:

Effort level of B	1	2	3	4	5	6	7	8	9	10
Your income (in points)	1	16	29	41	53	64	75	82	87	90
Income of B (in points)	99	98	96	93	89	83	75	65	51	35

Please answer the questions.

The experiment: decide!

INSTRUCTIONS

You can force participant B to exert a minimum effort level of 1, 2, or 3. At the same time, participant B chooses an effort level from each of the following three tables. Your decision determines which of the three decisions of B will be relevant for calculating the incomes.

Please choose one of the following three options.

Which minimum effort level do you enforce from participant B?

○ **Option 1**: I force B to exert an effort level of at least 1.

Effort level of B	1	2	3	4	5	6	7	8	9	10
Your income (in points)	1	16	29	41	53	64	75	82	87	90
Income of B (in points)	99	98	96	93	89	83	75	65	51	35

○ **Option 2**: I force B to exert an effort level of at least 2.

Effort level of B	1	2	3	4	5	6	7	8	9	10
Your income (in points)		16	29	41	53	64	75	82	87	90
Income of B (in points)		98	96	93	89	83	75	65	51	35

○ **Option 3**: I force B to exert an effort level of at least 3.

Effort level of B	1	2	3	4	5	6	7	8	9	10
Your income (in points)			29	41	53	64	75	82	87	90
Income of B (in points)			96	93	89	83	75	65	51	35

Please answer the question.

The experiment: make a guess!

INSTRUCTIONS

Assume there are 100 participants A (the actual number of participants A will be converted to 100). What do you guess, how many participants A will decide on the options 1, 2, and 3 in this round? If your guess does not differ by more than 5 from the converted number of participants A who actually decide on a given option then you will receive **70 points**. Otherwise you will receive 20 points.

No other participant will ever know your answer. Please always insert an integer without decimals. Note that the three numbers have to sum up to 100.

I guess out of 100 participants A, ...

Option 1: about [ ] **force** their participant B to exert an effort level of **at least 1**.

Option 2: about [ ] **force** their participant B to exert an effort level of **at least 2**.

Option 3: about [ ] **force** their participant B to exert an effort level of **at least 3**.

The general income table is shown once more for visualization:

Your effort level	1	2	3	4	5	6	7	8	9	10
Income of A (in points)	1	16	29	41	53	64	75	82	87	90
Your income (in points)	99	98	96	93	89	83	75	65	51	35

Please answer the questions.

The experiment: decide!

INSTRUCTIONS

Participant A decides whether to force you to exert an effort level of at least 1, 2, or 3 (without knowing what you decide). Since you do not yet know the decision of A you have to choose an effort level for each of the three cases. The decision of A determines which of your three decisions will be relevant for calculating the incomes.

In each table, click on the column of the effort level you want to choose.

Which effort level do you choose?

Case 1: Assume that A forces you to exert an effort level of at least 1.

Your effort level	1	2	3	4	5	6	7	8	9	10
Income of A (in points)	1	16	29	41	53	64	75	82	87	90
Your income (in points)	99	98	96	93	89	83	75	65	51	35

Case 2: Assume that A forces you to exert an effort level of at least 2.

Your effort level	1	2	3	4	5	6	7	8	9	10
Income of A (in points)		16	29	41	53	64	75	82	87	90
Your income (in points)		98	96	93	89	83	75	65	51	35

Case 3: Assume that A forces you to exert an effort level of at least 3.

Your effort level	1	2	3	4	5	6	7	8	9	10
Income of A (in points)			29	41	53	64	75	82	87	90
Your income (in points)			96	93	89	83	75	65	51	35

Please answer the questions.

The experiment: incomes

You are participant A.

The decisions of this round are shown in the following table:

**Effort level of B**	1	**2**	**3**	**4**	**5**	**6**	**7**	**8**	**9**	**10**
Your income (in points)		16	29	41	53	64	75	82	87	90
Income of B (in points)		98	96	93	89	83	75	65	51	35

You have decided to force participant B to exert an effort level of at least 2. In this case, participant B has chosen an effort level of 4. Your income is 41 points and the income of participant B is 93 points.

Just to remind you: **Only one round will be selected for payment.** For the selected round, **either** your income from the decisions **or** your income from one of the three guesses will be selected for payment. Which round and which income of the respective round is relevant for payment will be selected at random after the experiment is over.

If the **income from the decisions of this round is selected for payment** then **your income** will be converted to **6.15 euros** and the income of participant B will be converted to 13.95 euros.

The next round will start soon. 24s

The experiment: incomes

You are participant B.

The decisions of this round are shown in the following table:

**Your effort level**	1	**2**	**3**	**4**	**5**	**6**	**7**	**8**	**9**	**10**
Income of A (in points)		16	29	41	53	64	75	82	87	90
Your income (in points)		98	96	93	89	83	75	65	51	35

Participant A has decided to force you to exert an effort level of at least 2. In this case, you have chosen an effort level of 4. The income of participant A is 41 points and your income is 93 points.

Just to remind you: **Only one round will be selected for payment.** For the selected round, **either** your income from the decisions **or** your income from one of the three guesses will be selected for payment. Which round and which income of the respective round is relevant for payment will be selected at random after the experiment is over.

If the **income from the decisions of this round is selected for payment** then the income of participant A will be converted to 6.15 euros and **your income** will be converted to **13.95 euros**.

The next round will start soon. 29s

D.2 Handling troubles

Now we briefly explain typical troubles that occurred in the study and our solutions.

Late comers. Participants who logged in to the experiment after the session had already started were excluded.

Odd number of participants. If the number of remaining participants was odd after the control questions, i.e. before roles were assigned, a randomly selected participant was excluded from the session.

Time limit (nearly) exceeded. To synchronize participants, we divided the experiment into several blocks with timeouts. Participants for whom the timeout of a given block had nearly elapsed were informed of the remaining time. Participants who failed to complete the block before the timeout were excluded from further participation.

Participant re-enters. On the internet it can well happen that a browser crashes or is closed by accident. In such cases participants could login again, but the time needed to complete the block properly was computed (taking the read mode into account) and compared to the time remaining until timeout. Depending on whether the remaining time was sufficient or not, the participant could either continue or was excluded.

Partner dropped out. In particular, participants who drop disturb the regular course of the experiment for their interaction partners. Participants without a partner after a given round were excluded from the experiment. Solutions to dropouts during rounds were implemented in the matching protocol as illustrated in Figure 2.

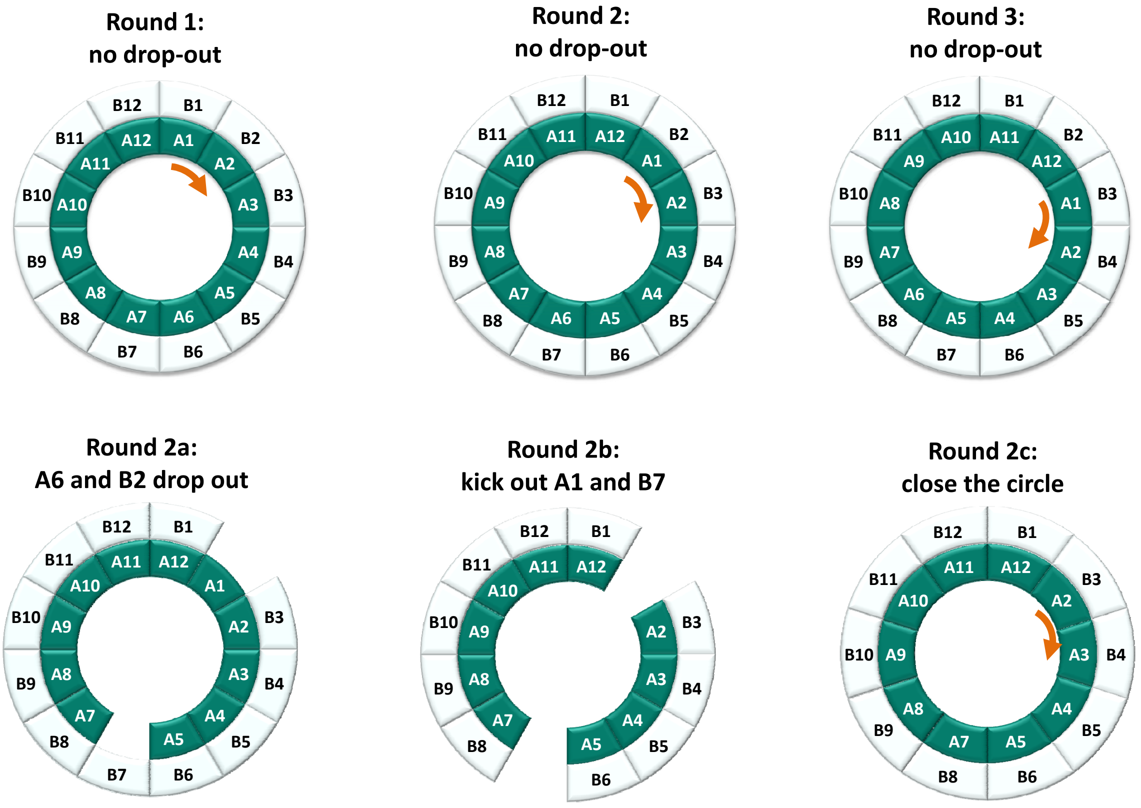

Figure 2: “No-contagion” matching with(out) dropouts.

Note: The figure illustrates the matching for 24 participants, 12 of them in the role of A and 12 in the role of B. The upper (lower) panel illustrates the matching protocol without (with) dropouts.

E Comparing data from Jena and Konstanz

Our data were collected in two locations. Four sessions (two in the laboratory and two on the internet) were conducted in Jena (2010/11) and twelve sessions (six sessions per experiment) were conducted in Konstanz (2014/15). This section reports a series of statistics comparing the data from both locations.

As detailed in the following sections, we have collected 42 p-values of tests comparing data from Jena and Konstanz. If the p-values were generated by chance, they should be uniformly distributed. If the data from Jena and Konstanz differed, we would observe more lower and less higher p-values. Less than half (19) of our p-values are smaller than 0.5. Moreover, 8 tests reject the null hypothesis that the data from the two locations do not differ at the 10 percent level, only 3 tests at the 5 percent level, and the null hypothesis is never rejected at the 1 percent level. Figure 3 shows that apparently, the cumulative distribution of our p-values is very close to the cumulative uniform distribution. Obviously, our p-values are not more often significant than expected by random draws (otherwise, the cumulative frequency of p-values would exceed the cumulative uniform distribution for values below 0.05). We conclude that the data from Jena and Konstanz do not differ more than chance would predict.

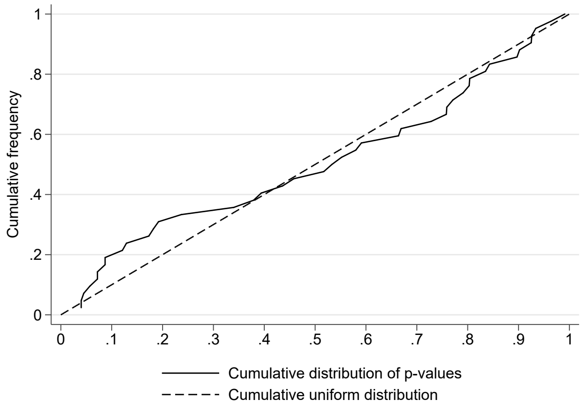

Figure 3: Cumulative distribution of p-values compared to the uniform distribution.

E.1 Agents' efforts across locations

Figure 4 shows for each experiment in each location efforts averaged across all agents and rounds. In the laboratory, agents in Konstanz appear to be more generous than agents in Jena. On the internet, effort levels are very similar in both locations. Since the main interest of this paper is not in absolute effort levels but in effort differences, level differences between the locations in the laboratory are not of a major concern. In both locations, we observe the same pattern: agents' efforts hardly differ between control levels in the laboratory while efforts increase with control on the internet.

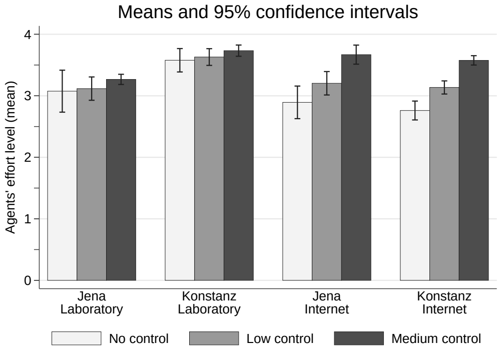

Figure 4: Average effort levels.

E.2 Agents' effort differences across locations

Table 4 reports four regression models as well as χ^2 tests to formally test whether we have to reject the null hypothesis that effort differences are the same in our two locations. We rely on linear mixed effects models including random intercepts at the agent and session levels.

	Dependent variable: Difference between effort under			
	low control and no control		medium and low control	
Model	(1)	(2)	(3)	(4)
Constant	0.052	0.115	0.104	0.112
	(0.115)	(0.121)	(0.107)	(0.111)
Int	0.322**	0.274	0.336**	0.310**
	(0.160)	(0.167)	(0.148)	(0.153)
Jena	-0.011	-0.157	0.046	0.071
	(0.240)	(0.251)	(0.222)	(0.230)
$Int * Jena$	-0.054	0.058	-0.021	-0.018
	(0.327)	(0.341)	(0.302)	(0.313)
$Half2$		-0.125*		-0.018
		(0.069)		(0.057)
$Int * Half2$		0.097		0.052
		(0.096)		(0.079)
$Jena * Half2$		0.292**		-0.049
		(0.145)		(0.119)
$Int * Jena * Half2$		-0.223		-0.006
		(0.197)		(0.161)
Observations	2,200	2,200	2,200	2,200
Log-likelihood	-3349.831	-3347.203	-2939.244	-2938.769
**Hypothesis testing**				
Laboratory: Jena = Konstanz				
Rounds 1-10	$p = 0.964$		$p = 0.835$	
Rounds 1-5		$p = 0.533$		$p = 0.758$
Rounds 6-10		$p = 0.591$		$p = 0.925$
Internet: Jena = Konstanz				
Rounds 1-10	$p = 0.771$		$p = 0.902$	
Rounds 1-5		$p = 0.669$		$p = 0.803$
Rounds 6-10		$p = 0.897$		$p = 0.991$

Notes: Standard errors in parentheses. ***(1%); **(5%); *(10%) significance level.

Table 4: Effort differences in both locations.

In Models 1 and 3, effort differences are regressed against an intercept, the experimental condition (where the dummy variable *Int* identifies data from the internet experiment), the location (where the dummy variable *Jena* identifies data from Jena) and their interaction. In models 2 and 4, a dummy *Half2* for the second half of rounds and its interactions with the experiment and location are added. For each experimental condition, we test whether effort differences in Jena and Konstanz differ in all 10 rounds, as well as in the first half (rounds 1-5) and the second half (rounds 6-10) of the experiment. The null hypothesis that effort differences in Jena and Konstanz do not differ is never rejected at the 10 percent level (χ^2 tests: $p - values > 0.1$).

We conclude that effort differences are similar in the two experiments in Jena and Konstanz and proceed with the pooled data set to test our first hypothesis.

E.3 Agents' beliefs across locations

Table 5 reports statistics on distributions of agents' beliefs in both locations and experiments. In each panel, the first row reports agents' average beliefs and the second row reports standard deviations. P-values report significance levels of Wilcoxon rank-sum tests of equal beliefs distributions in Jena and Konstanz for a given experiment and control level.[4] The upper part of the table considers all rounds while the lower part is restricted to experienced agents (rounds 6 to 10).

	Laboratory			Internet		
	Jena	Konstanz	$P-value$	Jena	Konstanz	$P-value$
Rounds 1-10						
$b_A(1)$	9.79	13.15	0.394	13.85	14.37	0.934
	(8.30)	(12.95)		(11.77)	(12.61)	
$b_A(2)$	12.49	18.00	0.057	19.22	18.87	0.804
	(10.16)	(12.69)		(11.14)	(12.49)	
$b_A(3)$	77.72	68.85	0.072	66.93	66.76	0.843
	(16.01)	(21.26)		(20.40)	(22.91)	
Rounds 6-10						
$b_A(1)$	8.43	12.02	0.552	11.84	13.13	0.759
	(8.67)	(14.93)		(13.43)	(13.78)	
$b_A(2)$	10.26	15.52	0.040	14.59	16.59	0.517
	(10.49)	(13.15)		(12.02)	(13.13)	
$b_A(3)$	81.31	72.46	0.087	73.57	70.28	0.664
	(17.38)	(22.53)		(21.54)	(23.86)	
Observations	24	80		29	87	

Notes: All $p-values$ rely on two-sample Wilcoxon rank-sum tests to test the null hypothesis of equal distributions in the two locations.

Table 5: Detailed statistics on agents' beliefs in both locations.

In both locations and experiments, agents expect little effort discretion and they expect slightly higher effort control over time. Agents' beliefs appear very similar in both locations on the internet and in the laboratory experiment in Konstanz, whereas agents in the laboratory in Jena seem to expect higher effort control than all other agents.

In the laboratory, we do not reject the null hypothesis that the distribution of beliefs, averaged across all rounds for each agent, concerning the frequency of principals that choose no, low or medium control is the same in Jena and Konstanz at the 5 percent level (Wilcoxon rank-sum tests: $p-values > 0.05$). However, laboratory agents in Jena expect the low (medium) control level weakly significantly less (more) often than their counterparts in Konstanz. The same tendency applies to beliefs of experienced agents. We do not reject the null hypothesis that the distribution of beliefs, averaged across rounds 6 to 10 for each agent, concerning no or medium control is the same in Jena and Konstanz at the 5 percent level (Wilcoxon rank-sum tests: $p-values > 0.05$). Experienced laboratory agents in Jena expect low control significantly less often than those in Konstanz, and the difference regarding medium control is again weakly significant. None of these significant effects survives the Bonferroni correction to account for multiple comparisons.

On the internet, the distributions of agents' beliefs are very similar in both locations. We do not reject the null hypothesis that the distribution of beliefs concerning no, low or medium control is the same in Jena and Konstanz at the 10 percent level (Wilcoxon rank-sum tests: $p-values > 0.1$). This is also true for the second half of rounds (Wilcoxon rank-sum tests: $p-values > 0.1$).

We conclude that agents' beliefs are rather similar in both locations within each experiment.

[4] As our Jena and Konstanz samples are independent, we rely on two-sample Wilcoxon rank-sum tests of the null hypothesis that distributions of agents' beliefs are the same in the two locations within each experiment. We average beliefs across rounds for each agent for a given control level. Since agents are not informed of the correctness of their beliefs, we treat individuals as independent observations with respect to their beliefs.

E.4 Principals' choices across locations

Table 6 summarizes principals' control decisions in the two experiments in Jena and Konstanz. In both experimental conditions, principals in Jena tend to control somewhat more than in Konstanz. This tendency is minor on the internet, and it is more pronounced in the laboratory.

	No control	Low control	Medium control
Jena Internet	14%	13%	73%
Konstanz Internet	16%	15%	69%
Jena Laboratory	13%	10%	77%
Konstanz Laboratory	15%	20%	65%

Table 6: Frequencies of principals' control levels in both locations.

Table 7 reports two regression models as well as χ^2 tests to formally test whether we have to reject the null hypothesis that control levels are the same in our two locations. We rely on linear mixed effects models including random intercepts at the principal and session levels.

The null hypothesis that control levels in Jena and Konstanz do not differ is never rejected at the 10 percent level (χ^2 tests: $p-values > 0.1$). We conclude that control levels are similar for both experiments in Jena and Konstanz and pool principals' choices from both locations to test our second hypothesis.

	Dependent variable: Level of control	
Model	(1)	(2)
Constant	2.492***	2.425***
	(0.055)	(0.059)
Int	0.035	0.000
	(0.076)	(0.081)
$Jena$	0.153	0.117
	(0.115)	(0.122)
$Int * Jena$	-0.095	0.030
	(0.156)	(0.166)
$Half2$		0.135***
		(0.041)
$Int * Half2$		0.070
		(0.056)
$Jena * Half2$		0.073
		(0.085)
$Int * Jena * Half2$		-0.250**
		(0.115)
Observations	2,200	2,200
Log-likelihood	-2144.184	-2121.087
**Hypothesis testing**		
Laboratory: Jena = Konstanz		
Rounds 1-10	$p = 0.182$	
Rounds 1-5		$p = 0.340$
Rounds 6-10		$p = 0.121$
Internet: Jena = Konstanz		
Rounds 1-10	$p = 0.580$	
Rounds 1-5		$p = 0.192$
Rounds 6-10		$p = 0.791$

Notes: Standard errors in parentheses.
***(1%); **(5%); *(10%) significance level.

Table 7: Control intensity in both locations.

E.5 Principals' beliefs across locations

Table 8 reports statistics on distributions of principals' beliefs for each control level in both locations and experiments. In each panel and for each experiment, the first row reports principals' average beliefs and the second row reports standard deviations. P-values report significance levels of Wilcoxon rank-sum tests of equal beliefs distributions in Jena and Konstanz for a given experiment and control level. As our Jena and Konstanz samples are independent, we rely on two-sample Wilcoxon rank-sum tests of the null hypothesis that distributions of principals' beliefs are the same in the two locations within each experiment.[5] The upper part of the table considers all rounds while the lower part is restricted to experienced principals.

	Laboratory			Internet		
	Jena	Konstanz	$P-value$	Jena	Konstanz	$P-value$
Rounds 1-10						
$b_P(1)$	2.56	3.04	0.072	2.46	2.62	0.436
	(1.59)	(1.50)		(1.13)	(1.14)	
$b_P(2)$	3.12	3.57	0.087	3.21	3.17	0.926
	(0.91)	(1.15)		(0.99)	(0.86)	
$b_P(3)$	3.73	4.10	0.173	3.85	3.81	0.728
	(0.54)	(1.05)		(0.68)	(0.68)	
Rounds 6-10						
$b_A(1)$	2.30	2.82	0.040	2.25	2.34	0.379
	(1.67)	(1.57)		(1.41)	(1.12)	
$b_A(2)$	2.94	3.41	0.045	2.90	2.97	0.237
	(0.88)	(1.17)		(1.05)	(0.81)	
$b_A(3)$	3.65	3.97	0.129	3.64	3.72	0.458
	(0.59)	(1.01)		(0.73)	(0.74)	
Observations	24	80		29	87	

Notes: All $p-values$ rely on two-sample Wilcoxon rank-sum tests to test the null hypothesis of equal distributions in the two locations.

Table 8: Detailed statistics on principals' beliefs in both locations.

In the laboratory, we do not reject the null hypothesis that the distribution of beliefs, averaged across rounds for each principal, concerning the efforts that agents choose in case of no, low or medium control is the same in Jena and Konstanz at the 5 percent level (Wilcoxon rank-sum test: $p-values > 0.05$). However, laboratory principals in Jena always expect lower average efforts than their counterparts in Konstanz and these differences are weakly significant for the no and low control levels. The same tendency applies to beliefs of experienced principals. Expected efforts under medium control, averaged across rounds 6 to 10 for each principal, do not differ significantly between Jena and Konstanz at the 10 percent level (Wilcoxon rank-sum tests: $p-value > 0.1$), while experienced principals in the laboratory in Jena expect significantly lower average efforts under no and low control than those in Konstanz (Wilcoxon rank-sum tests: $p-values < 0.05$). None of these significant effects survives the Bonferroni correction to account for multiple comparisons.

On the internet, the distributions of principals' beliefs are very similar in both locations. We do not reject the null hypothesis that the distribution of beliefs is the same in Jena and Konstanz at the 10 percent level (Wilcoxon rank-sum tests: $p-values > 0.1$). Differences between principals' beliefs in the two locations are also non-significant in the second half of rounds on the internet (Wilcoxon rank-sum test: $p-values > 0.1$).

We conclude that principals' beliefs are rather similar in both locations within each experiment.

[5] As our Jena and Konstanz samples are independent, we rely on two-sample Wilcoxon rank-sum tests of the null hypothesis that distributions of principals' beliefs are the same in the two locations within each experiment. We average beliefs across rounds for each principal for a given control level. Since principals are not informed of the correctness of their beliefs, we treat individual principals as independent observations with respect to their beliefs.

F Complementary data analysis

F.1 Cumulative frequencies of agents' effort

Figure 5 shows the cumulative frequencies of agents' effort per control level. We observe that under no and low control efforts lower than six are made more often in the internet than in the laboratory whereas the cumulative distribution of efforts under medium control is hardly distinguishable between the two treatments.

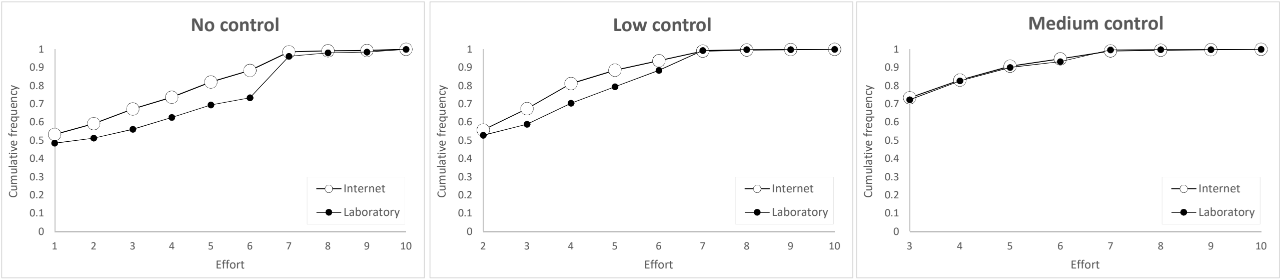

Figure 5: Cumulative frequency of efforts per control level.

F.2 Agents' effort over time

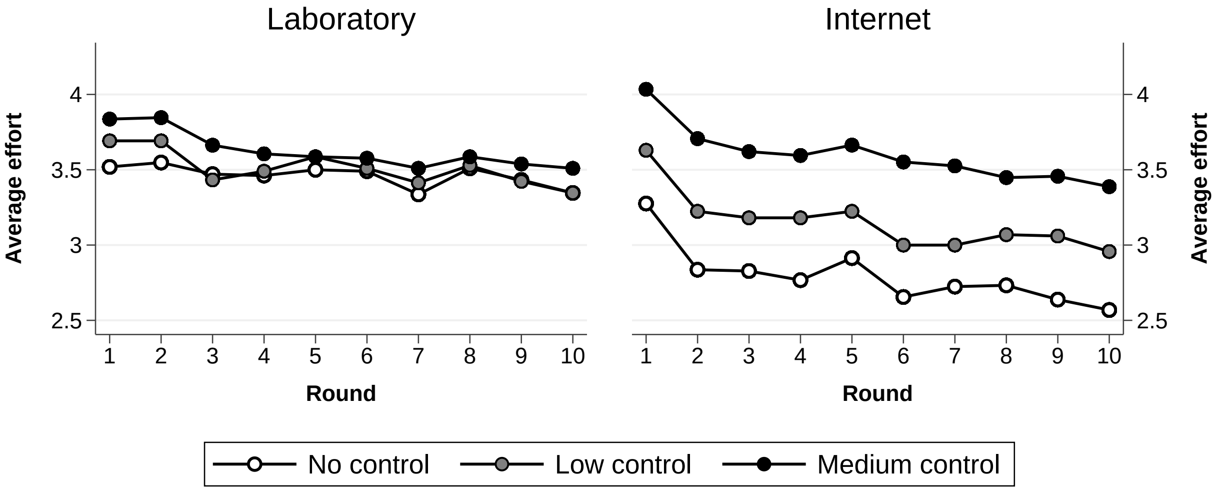

Figure 6: Average effort over time.

Figure 7 shows the relative frequency of each effort level chosen by agents in case of no, low and medium control over time. Grayish colors represent choices at the three enforcement levels, greenish colors reflect prosocial choices beyond the highest enforcement level and below the fair effort level, which is colored in blue. Finally, effort levels which leave the agent with a disadvantageous payoff compared to the principal are represented by reddish colors.

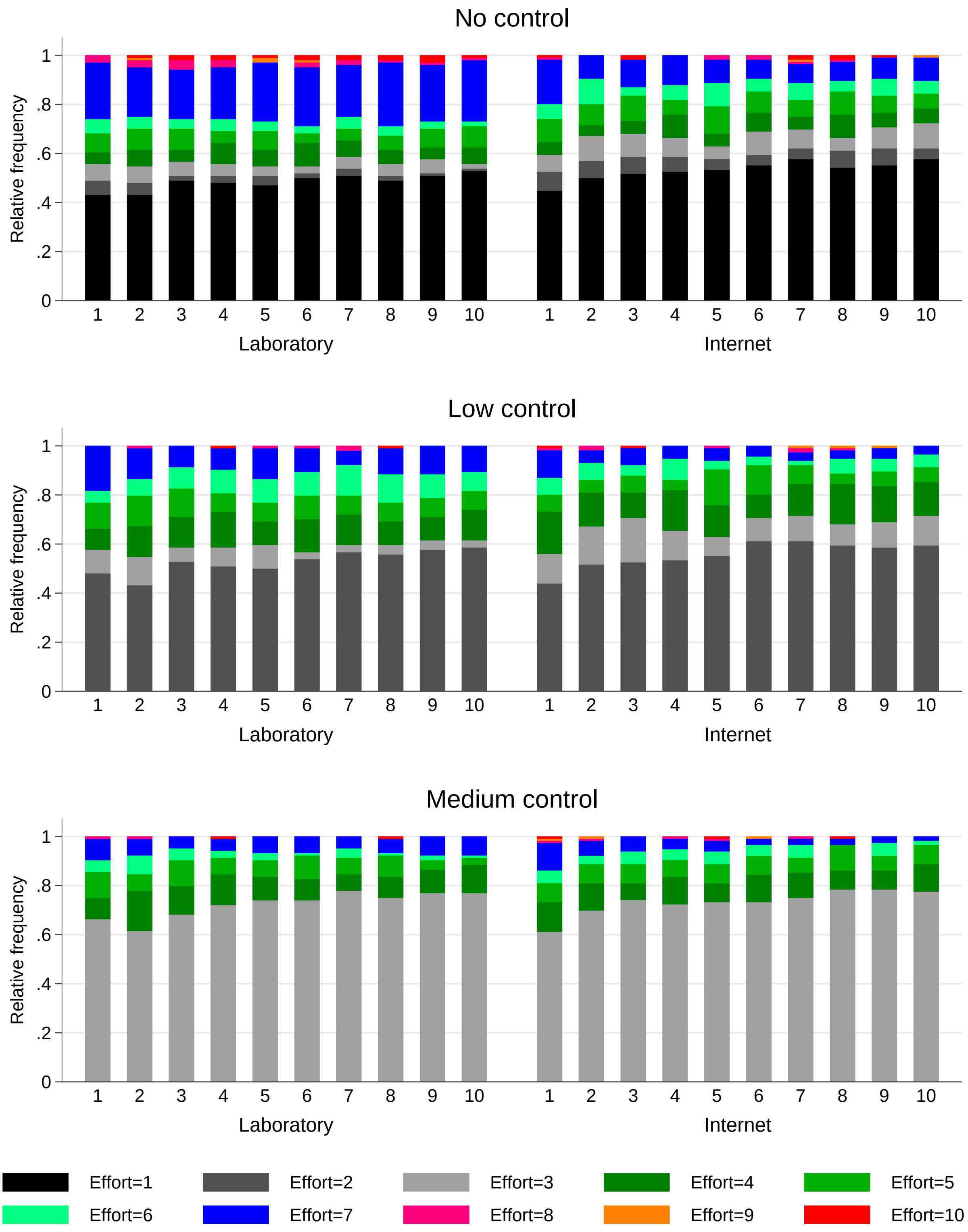

Figure 7: Frequency of effort levels over time.

F.3 Dynamics of the reactions to (the absence of) control and agents' types

In a given round, the triplet of effort levels $(e(1), e(2), e(3))$ captures the agent's reactions to (the absence of) control. These reactions can be partitioned into four qualitatively distinct categories that are defined in Table 9.

Category	Description	Behavioral definition	
		Low vs. no control	Medium vs. low control
Selfish	Always chooses minimal effort	$e(1) = 1 \wedge e(2) = 2$	$e(2) = 2 \wedge e(3) = 3$
Control neutral	Does not react to control	$e(1) = e(2)$	$e(2) = e(3)$
Control averse	Motivational crowding out	$e(1) > e(2)$	$e(2) > e(3)$
Control liking	Motivational crowding in	$e(1) < e(2) \wedge e(2) > 2$	$e(2) < e(3) \wedge e(3) > 3$

Table 9: A qualitative classification of the reactions to (the absence of) control.

Figure 8 shows the frequencies of the reactions to (the absence of) control over time. The upper (lower) graph illustrates how agents react to low compared to no (medium compared to low) control. Reactions classified as selfish are always most frequent (around 50%) and tend to increase over time for both experimental conditions. Indirect positive reactions to control classified as control liking are very rare (less than 10%) and they almost disappear over time. The share of control neutral reactions is about 15% in both treatments and persists across all rounds. Most importantly, control averse reactions are robust over time and more frequent in the laboratory (they constitute 29% of all reactions in the laboratory and only 21% online).

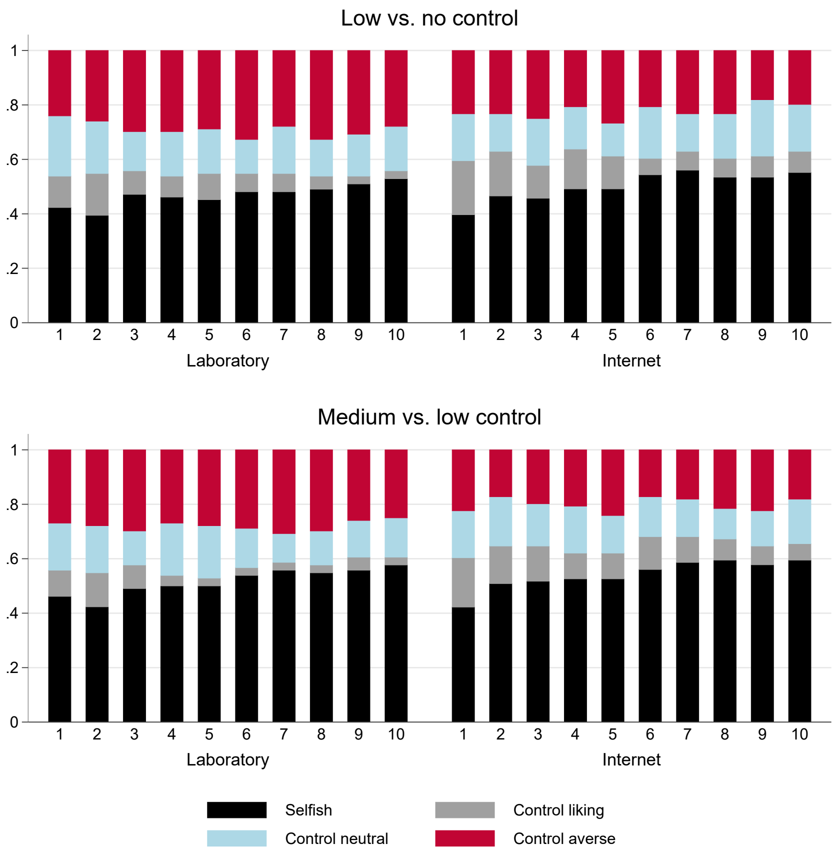

Figure 8: Frequency of reactions to (the absence of) control over time.

Agents' types

The type of selfish agents is always most common, fairly robust, and slightly more frequent online. Around 29% (25%) of agents in the Internet (laboratory) have selfish reactions in all 10 rounds, and about half of the agents are selfish in the majority of rounds (i.e., in at least 6 rounds). Yet, approximately a quarter of agents never behave as a selfish type.

Control aversion can also be associated with a relatively persistent type, and more so in the laboratory. Around 6% (10%) of agents in the Internet (laboratory) show control averse reactions in all 10 rounds, and about 17% (28%) in the Internet (laboratory) are control averse in the majority of rounds. About half of the agents express control aversion in at least one round.

The type of control neutral agents exists but is rather rare. Only 3% of agents always react neutrally to control, around 10% do so in the majority of rounds, while nearly half adopt such a reaction in at least one round. Both treatments are very similar with respect to the control neutral type.

The type of reactions we interpret as control liking is very rare and somewhat more frequent online. Around 66% (73%) of our Internet (laboratory) agents never show crowding in, less than 5% adopt such a reaction in the majority of rounds, and hardly any agent does so consistently across all rounds. We infer that this behavior appears to be rather noisy and cannot be associated with a stable type.

F.4 Agents' beliefs

Figure 9 shows for each treatment and in each round the beliefs of agents regarding the proportion of principals who choose either of the control levels.

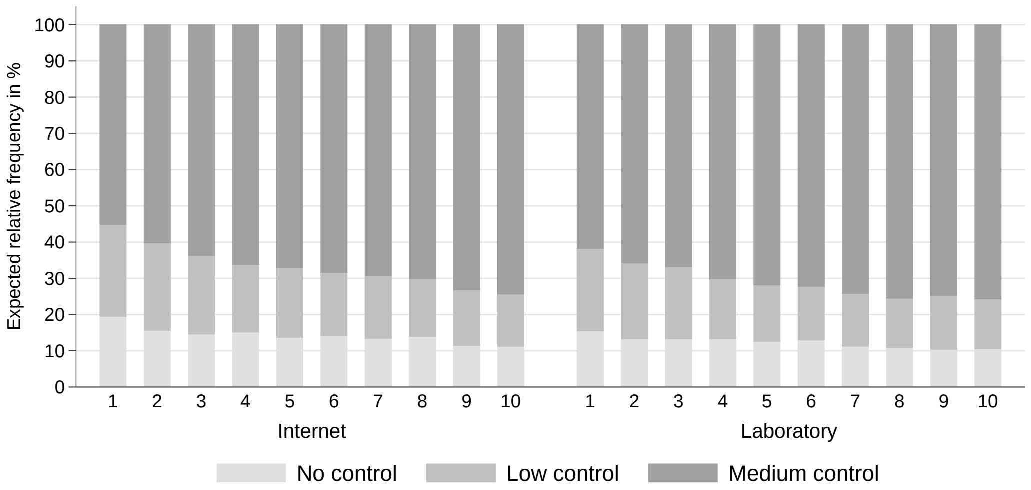

Figure 9: Agents' beliefs over time.

As our internet and laboratory samples are independent, we rely on two-sample Wilcoxon rank-sum tests of the null hypothesis that distributions of agents' beliefs are the same in the two treatments. We average beliefs across rounds for each agent for a given control level.[6]

The upper three panels of Table 10 report statistics on distributions of agents' beliefs for each control level. In each panel and for each treatment, the first row reports agents' average beliefs and the second row reports standard deviation. P-values report significance levels of Wilcoxon rank-sum tests of equal beliefs distributions in the internet and laboratory treatments for a given control level. The part on the left of the table considers all rounds while the part on the right is restricted to experienced agents.

We approach the correctness of agents' beliefs with the help of mean squared deviations (MSD) from the relative frequencies of control levels chosen by the principals. For each agent, we compute the squared difference between the agent's belief and the relative frequency of each control level chosen by principals in a given treatment, and we average the mean squared deviations over control levels and rounds. Thus, for

[6]Based on the fact that agents are not informed of the correctness of their beliefs, we treat individual agents as independent observations with respect to their beliefs. Our conclusions do not change if we rely on the level of sessions as independent observations.

	Treatment			Treatment		
	Internet	Laboratory	$P - value$	Internet	Laboratory	$P - value$
Rounds used	$1-10$	$1-10$	$1-10$	$6-10$	$6-10$	$6-10$
$b_A(1)$	14.24	12.37	0.354	12.81	11.19	0.487
	(12.35)	(12.08)		(13.64)	(13.78)	
$b_A(2)$	18.95	16.73	0.135	16.09	14.31	0.281
	(12.12)	(12.33)		(12.84)	(12.73)	
$b_A(3)$	66.81	70.90	0.203	71.10	74.50	0.348
	(22.22)	(20.45)		(23.26)	(21.70)	
MSD	0.040	0.040	0.884	0.036	0.036	0.710
	(0.032)	(0.036)		(0.042)	(0.047)	
Observations	116	104		116	104	

Notes: All $p - values$ rely on two-sample Wilcoxon rank-sum tests to test the null hypothesis of equal distributions in the two treatments.

Table 10: Detailed statistics on agents' beliefs.

each treatment, we first generate a matrix of relative frequencies of control levels chosen by the principals for each round.[7] Now we calculate the mean squared deviations between agents' beliefs and the matrix. For each round, we elicited agents' beliefs about each choice level. This estimated frequency vector can be compared to the actual {1, 2, 3} choice vector in a given round to generate a squared deviation score for agent i in round t as follows:

$$MSD^t_{Ai} = \frac{1}{3}\sum_{\underline{e}=1}^{3}(b^t_{Ai}(\underline{e}) - f^t_P(\underline{e}))^2,$$

where $b^t_{Ai}(\underline{e})$ is the estimated relative frequency of no, low or medium enforcement for agent i in round t and $f^t_P(\underline{e})$ is the relative frequency of principals' actual choices in round t. For each treatment, we average these SD scores for each agent across all 10 rounds and across the final 5 rounds.

The bottom panel of Table 10 reports summary statistics and p-values with respect to distributions of mean squared differences between agents' beliefs and principals' actual choices.

F.5 Robustness check of hypothesis 2's test

In the main text, we test Hypothesis 2 with the help of linear mixed models which implicitly assume that principals' choices are measured on a continuous scale. However, principals choose their levels of control from three ordered categories (no control, low control, and medium control). We check the robustness of the results reported in the main text by estimating regression models that account for the ordinal nature of the dependent variable. These ordered probit models have the same specifications as the main text models and they also include random intercepts at the principal and session levels. Table 11 reports the robustness results which confirm the main text findings: (1) Principals do not enforce more effort on the internet than in the laboratory; (2) Experienced principals control more; and (3) The more principals expect an increase in agents' effort due to an increase in the level of control, the higher the control level they choose.

F.6 Principals' beliefs

We test the similarity of principals' beliefs distributions in our two treatments with the help of two-sample Wilcoxon rank-sum tests. For each principal and each control level, we average beliefs with respect to agents' effort levels across rounds.[8]

[7] Note that we rely on choices of principals who completed the experiment, excluding those who dropped out. We obtain very similar results when also including the choices of principals who dropped out, and when computing MSD on the level of sessions instead of treatments.

[8] Based on the fact that principals are not informed of the correctness of their beliefs, we treat individual principals as independent observations with respect to their beliefs. Our conclusions do not change fundamentally if we rely on the level of sessions as independent observations.

	Dependent variable: Level of control				
Model	(1)	(2)	(3)	(4)	(5)
Int	0.021	-0.000	-0.037	-0.044	-0.062
	(0.166)	(0.178)	(0.166)	(0.167)	(0.168)
$Half2$		0.380***	0.335***	0.259***	0.262***
		(0.090)	(0.091)	(0.099)	(0.099)
$Int * Half2$		0.060	0.014	-0.034	-0.036
		(0.124)	(0.126)	(0.147)	(0.147)
$b_P(2) - b_P(1)$			0.227***	0.196***	0.196***
			(0.045)	(0.056)	(0.056)
$b_P(3) - b_P(2)$			0.274***	0.223***	0.220***
			(0.050)	(0.060)	(0.060)
$Int * [b_P(2) - b_P(1)]$			0.074	0.075	0.075
			(0.062)	(0.074)	(0.074)
$Int * [b_P(3) - b_P(2)]$			-0.033	-0.011	-0.010
			(0.066)	(0.078)	(0.078)
$Half2 * [b_P(2) - b_P(1)]$				0.074	0.072
				(0.081)	(0.081)
$Half2 * [b_P(3) - b_P(2)]$				0.127	0.125
				(0.083)	(0.083)
$Int * Half2 * [b_P(2) - b_P(1)]$				0.042	0.042
				(0.119)	(0.119)
$Int * Half2 * [b_P(3) - b_P(2)]$				-0.011	-0.010
				(0.118)	(0.118)
Age					0.084
					(0.157)
$Male$					0.245
					(0.166)
$Social$					-0.066
					(0.194)
Hum					-0.013
					(0.218)
$Tech$					-0.071
					(0.235)
Observations	2,200	2,200	2,200	2,200	2,200
Log-likelihood	-1565.859	-1543.605	-1470.986	-1466.862	-1465.140

Notes: Standard errors in parentheses. ***(1%); **(5%); *(10%) significance level.

Table 11: Determinants of the control intensity (ordered probit models).

The upper three panels of Table 12 report statistics on distributions of principals' beliefs for each control level. In each panel and for each treatment, the first row reports principals' average expected effort level and the second row reports standard deviation. P-values report significance levels of Wilcoxon rank-sum tests of equal beliefs distributions in the internet and laboratory treatments for a given control level. The part on the left of the table considers all rounds while the part on the right is restricted to experienced principals.

The null hypotheses of equal beliefs distributions across control levels within each treatment ($H_0 : b_P(1) = b_P(2)$ and $H_0 : b_P(2) = b_P(3)$) are tested with Wilcoxon matched-pairs signed-rank tests and always rejected at the 1 percent level (omitted in Table 12, $p - values = 0.0000$).

Principals do not expect average efforts in the two treatments to significantly differ for no, low or medium control at the 5 percent level (Wilcoxon rank-sum tests: $p - value > 0.05$ in all three cases). Similar conclusions hold for the no and medium control levels if we restrict principals' beliefs to the second

	Treatment			Treatment		
	Internet	Laboratory	$P-value$	Internet	Laboratory	$P-value$
Rounds used	$1-10$	$1-10$	$1-10$	$6-10$	$6-10$	$6-10$
$b_P(1)$	2.58	2.93	0.218	2.32	2.70	0.092
	(1.13)	(1.53)		(1.19)	(1.60)	
$b_P(2)$	3.18	3.46	0.095	2.95	3.30	0.013
	(0.89)	(1.11)		(0.87)	(1.12)	
$b_P(3)$	3.82	4.02	0.195	3.70	3.90	0.066
	(0.68)	(0.97)		(0.73)	(0.94)	
MSD	2.08	2.57	0.110	1.48	2.40	0.000
	(1.70)	(2.59)		(1.74)	(3.20)	
Observations	116	104		116	104	

Notes: All $p-values$ rely on two-sample Wilcoxon rank-sum tests to test the null hypothesis of equal distributions in the two treatments.

Table 12: Detailed statistics on principals' beliefs.

half of rounds (Wilcoxon rank-sum tests: $p-values > 0.05$ in both cases). Note that, in absolute numbers, principals always expect higher average efforts in the laboratory than on the internet. This difference is significant only at the low control level in the final 5 rounds (Wilcoxon rank-sum test: $p-value = 0.013$).

We approach the accuracy of principals' beliefs with the help of mean squared deviations (MSD) from agents' actual effort levels. For each principal, we compute the squared difference between the principal's belief and the average effort level chosen by agents in a given treatment, and we average the mean squared deviations over control levels and rounds. Thus, for each treatment, we first generate a matrix of average effort levels chosen by agents for each control level and round. Now we calculate the mean squared deviations between principals' beliefs and the matrix. For each round, we elicited principals' beliefs about agents' average effort for each of the three control levels. This estimated effort vector can be compared to the actual effort vector in a given round to generate a squared deviation score for principal i in round t as follows:

$$MSD_{Pi}^{t} = \frac{1}{3}\sum_{\underline{e}=1}^{3}(b_{Pi}^{t}(\underline{e}) - \overline{e}_{A}^{t}(\underline{e}))^2,$$

where $b_{Pi}^{t}(\underline{e})$ is the estimated average effort for no, low or medium enforcement for principal i in round t and $\overline{e}_{A}^{t}(\underline{e})$ is the agents' actual average effort in round t. For each treatment, we average these SD scores for each principal across all 10 rounds and across the final 5 rounds.

The bottom panel of Table 12 reports summary statistics and p-values with respect to distributions of mean squared differences between principals' beliefs and agents' actual choices. Aggregated across all rounds, the accuracy of principals' beliefs does not significantly differ in the two experiments (Wilcoxon rank-sum test: $p-value > 0.10$). However, principals' predictions seem to improve with experience on the internet. In the final 5 rounds, principals' beliefs differ highly significantly between the two experiments (Wilcoxon rank-sum test, $p-value < 0.001$). To understand why principals' expectations deviate less from agents' average efforts on the internet than in the laboratory, Table 13 again briefly summarizes principals' average beliefs and agents' average choices. Principals in both experiments expect efforts to increase with the level of control. Agents' average choices indeed increase with the level of control on the internet, but this is not true in the laboratory where average efforts hardly differ between control levels (see Figure 6 in Appendix F.2 and Table 4 in Section 3.2). Though principals tend to expect higher efforts in the laboratory than on the internet, they are still too pessimistic about agents' efforts in the absence of control in the laboratory experiment. It seems that principals do not anticipate control aversion and they are not aware of Frey's hypothesis.

F.7 Principals' monetary payoffs

Table 14 summarizes a series of regression models estimated by linear mixed effects models where random intercepts at the principal and the session level are included.

	Treatment		Treatment	
	Internet	Laboratory	Internet	Laboratory
Rounds used	1 − 10	1 − 10	6 − 10	6 − 10
$b_P(1)$	2.58	2.93	2.32	2.70
$b_P(2)$	3.18	3.46	2.95	3.30
$b_P(3)$	3.82	4.02	3.70	3.90
$\overline{e}_A(1)$	2.79	3.46	2.66	3.42
$\overline{e}_A(2)$	3.15	3.51	3.02	3.44
$\overline{e}_A(3)$	3.60	3.63	3.47	3.54

Table 13: Overview of principals' average beliefs and agents' average choices.

	Dependent variable: Principals' monetary payoff		
Model	(1)	(2)	(3)
Constant	35.244***	36.103***	36.799***
	(1.355)	(1.474)	(1.752)
Int	-0.907	-0.437	1.057
	(1.907)	(2.068)	(2.445)
$Half2$		-1.717	
		(1.161)	
$Int * Half2$		-0.940	
		(1.599)	
Round			-0.283
			(0.202)
$Int * Round$			-0.357
			(0.278)
Observations	2,200	2,200	2,200
Log-likelihood	-9585.080	-9581.074	-9578.519

Notes: Standard errors in parentheses.
***(1%); **(5%); *(10%) significance level.

Table 14: Determinants of the principals' monetary payoff.

According to model 1, principals' monetary payoffs are higher in the laboratory than in the internet treatment, but not significantly so. Payoffs are reduced over time and more so on the internet (model 2). In both halves of the treatment, differences in principals' payoffs between the laboratory and the internet are insignificant at the 10 percent level (χ^2 tests: $p - values > 0.10$). On the internet, principals earn significantly less in the second than in the first half of rounds (χ^2 test: $p - value = 0.016$). We draw very similar conclusions from model 3 where continuous time trends are captured by rounds and their interactions with the treatment.

F.8 Principals' best replies

Figure 10 shows for each treatment and in each round the proportion of principals who choose the control level which according to their elicited beliefs maximizes their monetary payoffs.

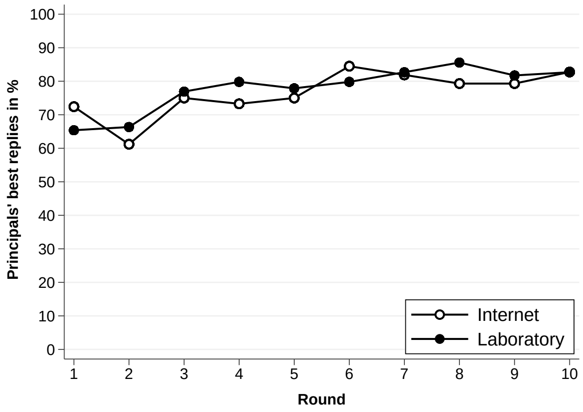

Figure 10: Frequencies of best-replies.

G Comparing the quality of laboratory and online data

Due to a lower degree of scrutiny than in the laboratory, data collected over the Internet might be of limited quality. We implemented a number of measures to minimize the difference in the data quality between the two treatments as detailed in Appendices B and D. In particular, experimental studies which compare data collected over the Internet and in the laboratory show that online decision times are lower than in the laboratory and that they are related to the pro-sociality of choices. This might be of concern for our study since the agent's intrinsic motivation plays a key role in the agency relationship. In an attempt to eliminate the confound of potentially different decision times, we implemented a read mode which prevents participants from just clicking through the screens. In the read mode, all active items like buttons or input elements were locked. Only after a few seconds, the screen switched into an edit mode where items became unlocked for participants to enter their decisions and continue. Of course, the read mode was implemented in both experiments.[9]

We compare the quality of laboratory and online data in several respects. First, we analyze whether online subjects had more difficulties to answer the control questions than laboratory subjects. We recorded the number of attempts a subject required to answer the control questions. In the laboratory (Internet) treatment, 64% (61%) of the subjects answered all questions correctly at the first attempt, only 7% (6%) had to try more than twice, and 1% (1%) failed to answer correctly even at the third attempt.[10] We fail to reject the null hypothesis that the distribution of the number of attempts is identical in the two treatments (Wilcoxon rank-sum test: $p - value = 0.524$). Second, we compare the frequency of confused choices in the two treatments. We postulate that an effort level chosen by the agent which gives her a lower monetary payoff than the principal indicates confusion (effort levels greater than the fair effort level of 7). Such choices are rare in each treatment. Considering all three choices in all ten rounds, 14 (13) agents made a confused choice at least once in the laboratory (Internet) treatment while 87% (89%) never chose an effort beyond 7. Our data do not suggest that confused choices occur more often online than in the laboratory and they are almost extinguished in the final round in each treatment (see Figure 7 in Appendix F.2). Third, we find that the variance of agents' choices under no and low control tends to be higher in the laboratory than online (as evident from Table 3 of the paper).

[9] On the decision and belief screens of round 1, the read mode lasted for 15 seconds and screens were unlocked after 5 seconds in later rounds.

[10] After three attempts subjects continued with the experiment even if they had not answered all questions correctly because we were reluctant to let our subject pool be harmed by frustration. After the third attempt two (three) subjects in the laboratory (Internet) treatment had not answered all questions correctly. Four of them answered at least four out of the six questions correctly and none of them got all questions wrong. The correct solutions were shown to subjects who had entered false answers.

Most online studies are implemented in a one-shot trial environment. By contrast, we implemented a repetitive trial environment which allows participants to gain experience with the interactive situation. We find that differences in agents' effort due to an increase in the level of control are larger online than in the laboratory and that these differences are stable over time and cannot be attributed to more confusion in the Internet treatment. We conjecture that in studies with potentially more noise in decision-making, due for example to more complex designs or non-student samples who have to get used to an abstract setting, repetitions are highly valuable as confusion is likely to reduce over time.

We conclude that thanks to a careful implementation of the agency relationship and the use of the same subject pool in the two treatments data of similar quality were collected over the Internet and in the laboratory.
